# Identification of diagnostic biomarkers via weighted correlation network analysis in colorectal cancer using a system biology approach

**DOI:** 10.1038/s41598-023-40953-5

**Published:** 2023-08-21

**Authors:** Soudeh Ghafouri-Fard, Arash Safarzadeh, Mohammad Taheri, Elena Jamali

**Affiliations:** 1 Men’s Health and Reproductive Health Research Center, Shahid Beheshti University of Medical Sciences, Tehran, Iran; 2 Department of Medical Genetics, Shahid Beheshti University of Medical Sciences, Tehran, Iran; 3 Institute of Human Genetics, Jena University Hospital, Jena, Germany; 4 Urology and Nephrology Research Center, Shahid Beheshti University of Medical Sciences, Tehran, Iran; 5 Department of Pathology, Loghman Hakim Hospital, Shahid Beheshti University of Medical Sciences, Tehran, Iran

**Keywords:** Cancer, Genetics, Molecular biology

## Abstract

Colorectal cancer (CRC) is the third most frequent cancer to be diagnosed in both females and males necessitating identification of effective biomarkers. An in-silico system biology approach called weighted gene co-expression network analysis (WGCNA) can be used to examine gene expression in a complicated network of regulatory genes. In the current study, the co-expression network of DEGs connected to CRC and their target genes was built using the WGCNA algorithm. GO and KEGG pathway analysis were carried out to learn more about the biological role of the DEmRNAs. These findings revealed that the genes were mostly enriched in the biological processes that were involved in the regulation of hormone levels, extracellular matrix organization, and extracellular structure organization. The intersection of genes between hub genes and DEmRNAs showed that DKC1, PA2G4, LYAR and NOLC1 were the clinically final hub genes of CRC.

## Introduction

Colorectal cancer (CRC) is the third most frequent cancer to be diagnosed in both females and males in the USA. Approximately 41% of CRC cases involve the proximal colon, whereas 22% and 28% involve the distal colon and rectum, respectively^1^. By 2030, there would be a 90% increase in the incidence rate of CRC in the USA^1^. People are keen to look for new treatment approaches in such a difficult environment^2^. CRC patients usually show no conventional clinical symptoms or just non-specific indicators in the early stage, which results in a low incidence of early diagnosis even though early diagnosis can greatly improve the prognosis^3^. Since it can be detected and effectively treated, CRC is regarded to be almost the ideal cancer for screening as it can be prevented from developing and causing both mortality and morbidity during its clinical course^4^.

An in-silico system biology approach called weighted gene co-expression network analysis (WGCNA) is used to examine gene expression in a complicated network of regulatory genes. This R-based tool uses genetic correlations to find modules that are highly connected. As a result, it is useful for discovering new cancer diagnostic and prognostic biomarkers^5–8^. Guo et al. found ten hub genes that could serve as viable biomarkers for clinical diagnosis and are associated with the progression of CRC^9^. In a separate study conducted by Cao et al., the hub genes TDRD5 and GPC1 were identified for prognosis prediction in CRC using WGCNA^10^. Using WGCNA, Lin et al. discovered that the complement and coagulation cascade, as well as the focal adhesion pathway, play a crucial role in the development and progression of CRC patients with liver metastasis. Additionally, they identified FGG, KNG1, CAV1, and SPP1 as potential metastatic markers that could aid in the early diagnosis of CRC^11^.

In the current study, the co-expression network of DEGs connected to CRC and their target genes was built using the WGCNA algorithm. This research will aid in the identification of viable biomarkers for CRC and further comprehension of the molecular mechanisms behind this cancer.

## Methods

### Microarray data acquisition

The following criteria were used to select all of the CRC datasets from the Gene Expression Omnibus (GEO): (1) microarray-based mRNA expression profiles could be accessible; and (2) patients with CRC and healthy controls were estimated. Consequently, a total of three microarray datasets (GSE141174 [Illumina humanRef-8 v2.0 expression beadchip], GSE184093 [Agilent-067406 Human CBC lncRNA + mRNA microarray V4.0 (Probe name version)] and GSE206800 [Clariom_D_Human] Affymetrix Human Clariom D Assay [transcript (gene) version]) were included in this study. We excluded lymph node samples from dataset GSE141174 and metastasis samples from dataset GSE206800. In this analysis, a total of 39 samples (16 controls and 23 cases) were examined details are provided in Table 1.

**Table 1:** Details of included datasets.

GEO accession number	Platform	Use	Patients	Controls	Tissue	Status
GSE141174	GPL6104	DEmRNA	8	8	Colon-Lymph node	Public on Feb 13, 2020
GSE184093	GPL20115GPL23126	DEmRNA	9	9	Colon	Public on Sep 17, 2021
GSE206800		DEmRNA	15	4	Colon	Public on Jun 26, 2022

### Microarray data analysis and identification of differentially expressed mRNAs

Each of the microarray datasets were normalized using the normalizeQuantiles function in the preprocessCore package (Version 1.58) and then merged. Considering that the Agilent and Affymertix platform datasets for this study were merged, the batch effect and technical variation were eliminated using ComBat function in sva package (version 3.44.0)^12^.

### Weighted correlation network analysis (WGCNA)

A scale-free network based on gene expression profiles was built using a systematic biological technique known as WGCNA. The R package WGCNA (version 1.72-1)^5^ was applied to create a weighted correlation network utilizing the mRNA expression profiles in the merged dataset in order to discover clinically meaningful modules of CRC. In summary, using the Pearson Correlation Coefficient test, the gene expression matrices were turned into matrices representing the similarities of paired mRNAs and then converted to adjacency matrices where the soft-threshold (power value) was applied to accentuate significant connections between genes and ignore weak correlations in the adjacency matrix. To represent the intensity of the connection between the genes, the adjacency matrix was then transformed into a topological overlap measure (TOM). Genes were analyzed via hierarchical clustering using TOM as an input, and network modules were found using the DynamicTreeCut function. Modules with high similarity scores were merged using a threshold value.

### Construction of module-trait relationships and identification of modules hub genes

Module Eigengene (ME) was used to describe expression profiles of each module as the eigenvector related to the first principal component of the expression matrix in order to discover modules that were strongly linked to the clinical variables that were being examined. Also, the correlations between specific genes and the CRC trait were assessed using the estimates of gene significance (GS). Additionally, the ME correlation and the gene expression profile for each module were defined as module membership (MM). The most significant (central) components of the modules can be said to be closely connected to the trait if the GS and MM have a substantial correlation. Lastly, we chose the GS and MM genes that showed a correlation of 0.7 or higher, and we considered the identical genes between the two groups as the most important genes.

### Protein–protein interaction network construction and hub clusters identification

The Search Tool for the Retrieval of Interacting Genes (STRING, https://string-db.org/) database was used to build the protein–protein interaction (PPI) network of the genes with highest GS and MM. Confidence score > 0.7 was set as significant. The hub clusters were chosen, and functional annotation was carried out, using the Cytoscape software plug-in molecular complex detection (MCODE)^13^.

### Gene ontology (GO) and KEGG pathway enrichment analysis of hub clusters

The biological process (BP), molecular function (MF), cellular component (CC), and KEGG pathway^14–16^ enrichment analysis of the mRNAs in the hub clusters were obtained utilizing ClusterProfiler R package (version 4.4.4)^17^. A criterion of an adjusted p value of 0.05 or less was established for the functional category.

### Identification of hub genes in PPI network using multiple centralities

By using maximal clique centrality (MCC) algorithm via cytohubba^18^ plugin in cytoscape, we discovered hub genes in the PPI network. Finally, the intersection of genes between hub genes and DEGs were identified as final hub genes.

### MiRNA- and TF-final hub genes regulatory networks

Using the NetworkAnalyst database^19^, connections between the final hub genes, transcription factors (TFs), and microRNAs (miRNAs) were established. The greatest degree in the networks was then found for each TF and miRNA.

### The final hub genes' genetic alterations in CRC patients

Using the cBioPortal for Cancer Genomics^20^, we examined the genetic changes of the final hub genes in CRC patients. We selected CRC datasets from the TCGA, which comprise 1510 CRC samples.

### Verification of final hub genes via expression values

The UALCAN^21^ and GSCALite databases^22^, a Web server for Gene Set Cancer Analysis, were used to confirm the mRNA expression patterns of the final hub genes in colorectal adenocarcinoma (COAD) and normal samples. Immunohistochemistry (IHC) from the Human Protein Atlas (HPA)^23^ was used to compare the protein expression of the final hub genes between COAD and normal tissues.

## Results

### Microarray data processing, integrative meta-analysis and assessment of data quality 

The role of mRNAs in CRC was examined utilizing three expression datasets that included samples from CRC and healthy control individuals. Using the normalizeQuantiles function in the preprocessCore package, quantile normalization was used to independently normalize each dataset. Then, gene symbols were used to combine three datasets. Next, the ComBat function from the R Package Surrogate Variable Analysis (SVA) was employed to rule out batch effects (non-biological differences). The raw data and normalized data following batch effect removal boxplots are displayed in Figure S1. These boxplots show a consistent level of quality in the expression data. Also, the boxplot of the preprocessed data showed satisfactory normalization. The PCA plot of all samples in the 2D plane included by their first two main components (PC1 and PC2) is shown in Figure S2 (PC1 and PC2). After batch effect reduction, the samples exhibited a fair dispersion, as seen by this plot.

### Identification of DEmRNAs for CRC

**Figure 1:**
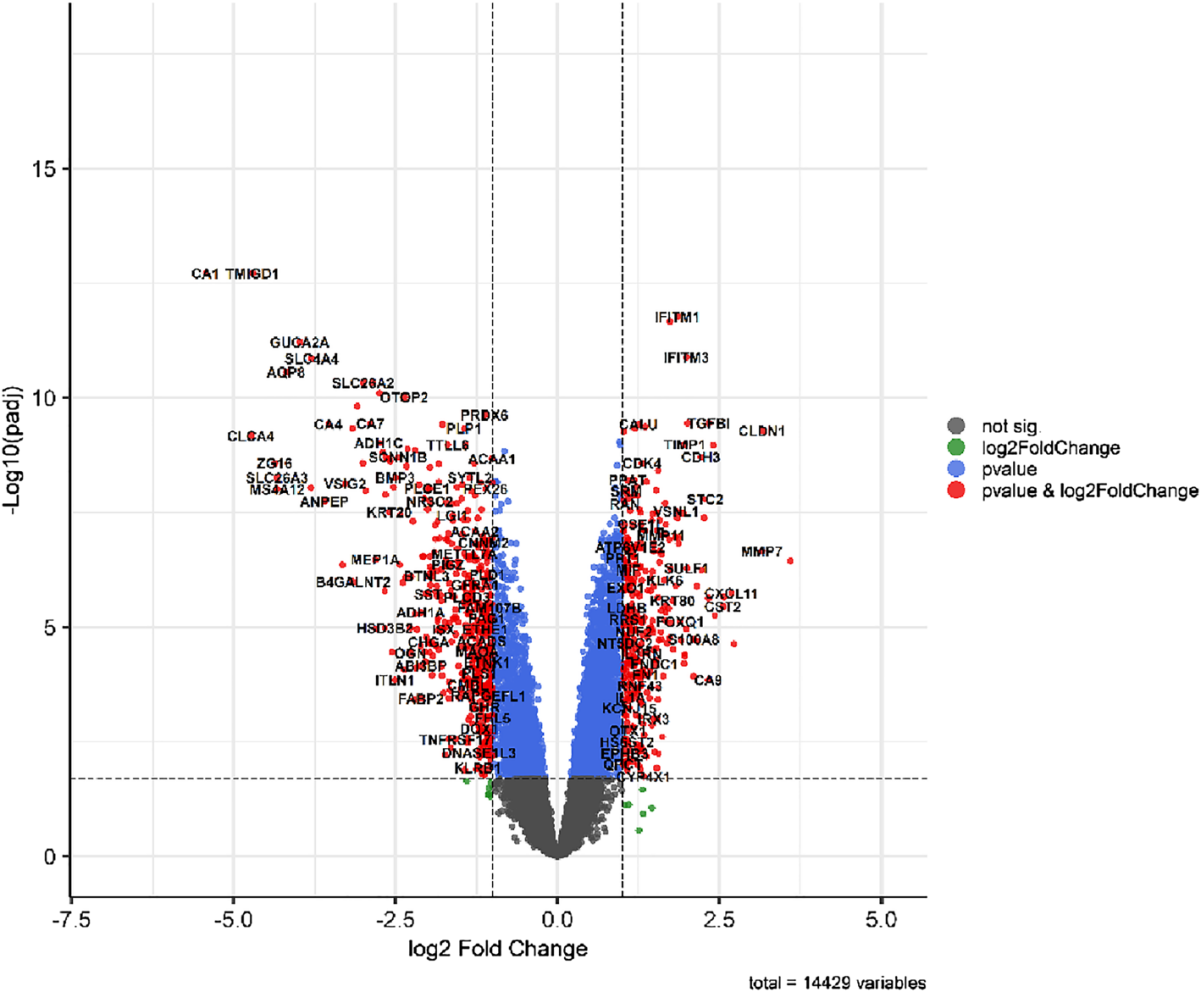
Volcano plot of DEGs; the horizontal axis represents the value of LogFC, while the vertical axis represents the mean value of -log 10 (false discovery rate). The significant dysregulated genes that fit the criteria are shown as red dots.

**Figure 2:**
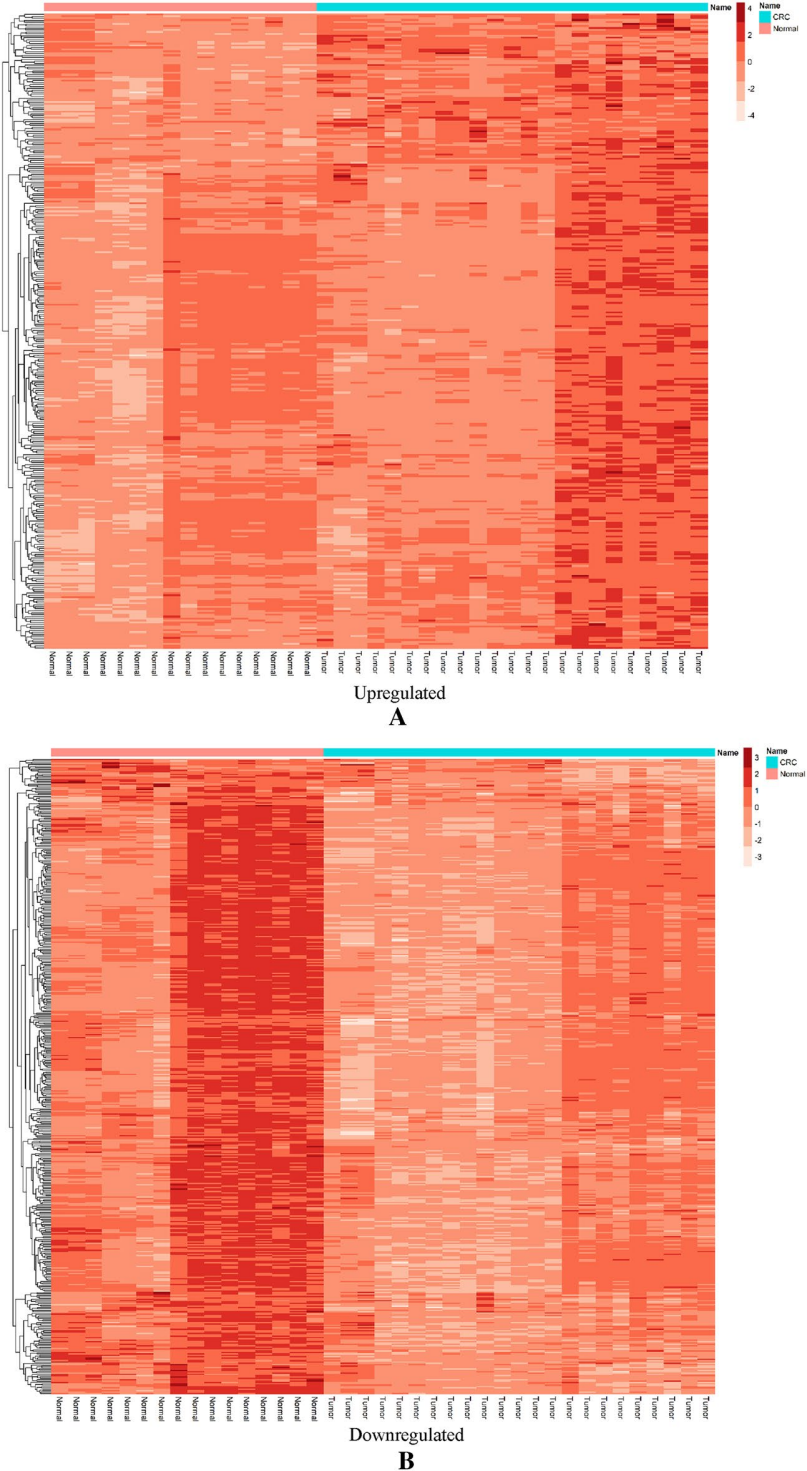
Differentially expressed mRNAs heatmaps. (**A**) Heatmap of upregulated DEmRNAs in CRC samples related to normal samples, (**B**) heatmap of downregulated DEmRNAs in CRC samples related to normal samples.

We analyzed the expression matrix between CRC and normal samples using the Limma package (version 3.52.3)^24^ and discovered 792 DEmRNAs, comprising 466 downregulated and 326 upregulated DEmRNAs (Table S1). Table 2 displays the top 10 DEmRNAs. The volcano plot was made using the EnhancedVolcano package (https://github.com/kevinblighe/EnhancedVolcano) in R, allowing us to examine the changes in mRNA expression between CRC and normal samples (Fig. 1). Also, we utilized the R pheatmap package (version 1.0.12) (https://cran.r-project.org/package=pheatmap) to show the expression of the top up- and down-regulated DEmRNAs (Fig. 2A,B).

**Table 2:** The top 10 up- and downregulated DEmRNAs between CRC and healthy control samples.

Down-regulated			Up-regulated		
DEmRNA	Log FC	Adjusted *P* value	DEmRNA	Log FC	Adjusted P value
CA1	− 5.432003828178	1.93031129373484e−13	CST1	3.597305699828	3.59334267864063e−07
CLCA4	− 4.736111996890	6.74412679226954e−10	CLDN1	3.160146475877	5.25282426604664e−10
TMIGD1	− 4.710900795511	1.93031129373484e−13	MMP7	3.155658772177	2.19314355683138e−07
ZG16	− 4.366573471589	2.64133741843165e−09	SPP1	2.727700004342	2.34022371696805e−05
SLC26A3	− 4.329846187037	5.48566830730311e−09	CXCL11	2.687229769694	1.81412617419494e−06
MS4A12	− 4.329097857632	9.73008672291592e−09	FAP	2.565935923642	2.1746407216848e−06
AQP8	− 4.194205533061	2.7357728781742e−11	CST2	2.551810987939	3.55596695929266e−06
GUCA2A	− 3.981801458697	6.11456410108153e−12	MMP3	2.426319689222	5.58503331923853e−06
CLCA1	− 3.808185178654	9.14732736571730e−09	CTHRC1	2.404456263036	1.07184057866666e−09
SLC4A4	− 3.797493894592	1.38388586917794e−11	TGFBI	2.332072366943	3.54833437548077e−10

### Gene Ontology (GO) and KEGG pathway enrichment analysis of DEmRNAs

**Figure 3:**
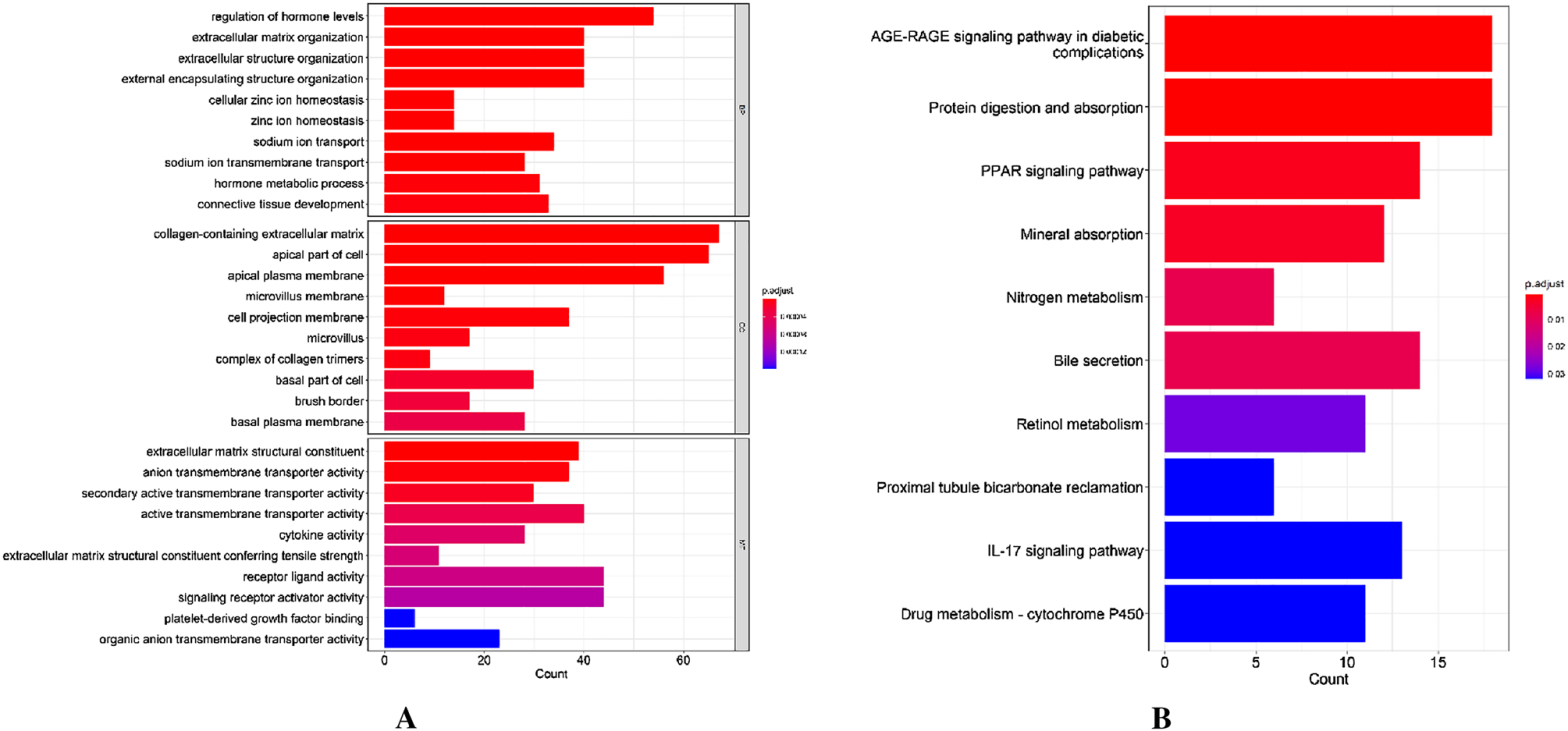
Results of DEmRNA analysis in Kyoto Encyclopedia of Genes and Genomes (KEGG) and the Gene ontology (GO). (**A**) Results of the investigation of DEmRNAs GO enrichment. The size of the bars corresponds to the gene number, while the color denotes the p value. (**B**) Results of DEmRNA KEGG pathway analysis. The size of the bars corresponds to the number of genes, while the color denotes the p value.

GO and KEGG pathway analysis were carried out to learn more about the biological role of the DEmRNAs. These findings revealed that the genes were mostly enriched in the BPs that were involved in the regulation of hormone levels, extracellular matrix organization, and extracellular structure organization (Fig. 3A). According the KEGG pathway, the AGE-RAGE signaling pathway in diabetic complications, protein digestion and absorption, and PPAR signaling pathway were the three main categories where the genes were enriched (Fig. 3B).

### Weighted correlation network analysis (WGCNA) and functional annotation

**Figure 4:**
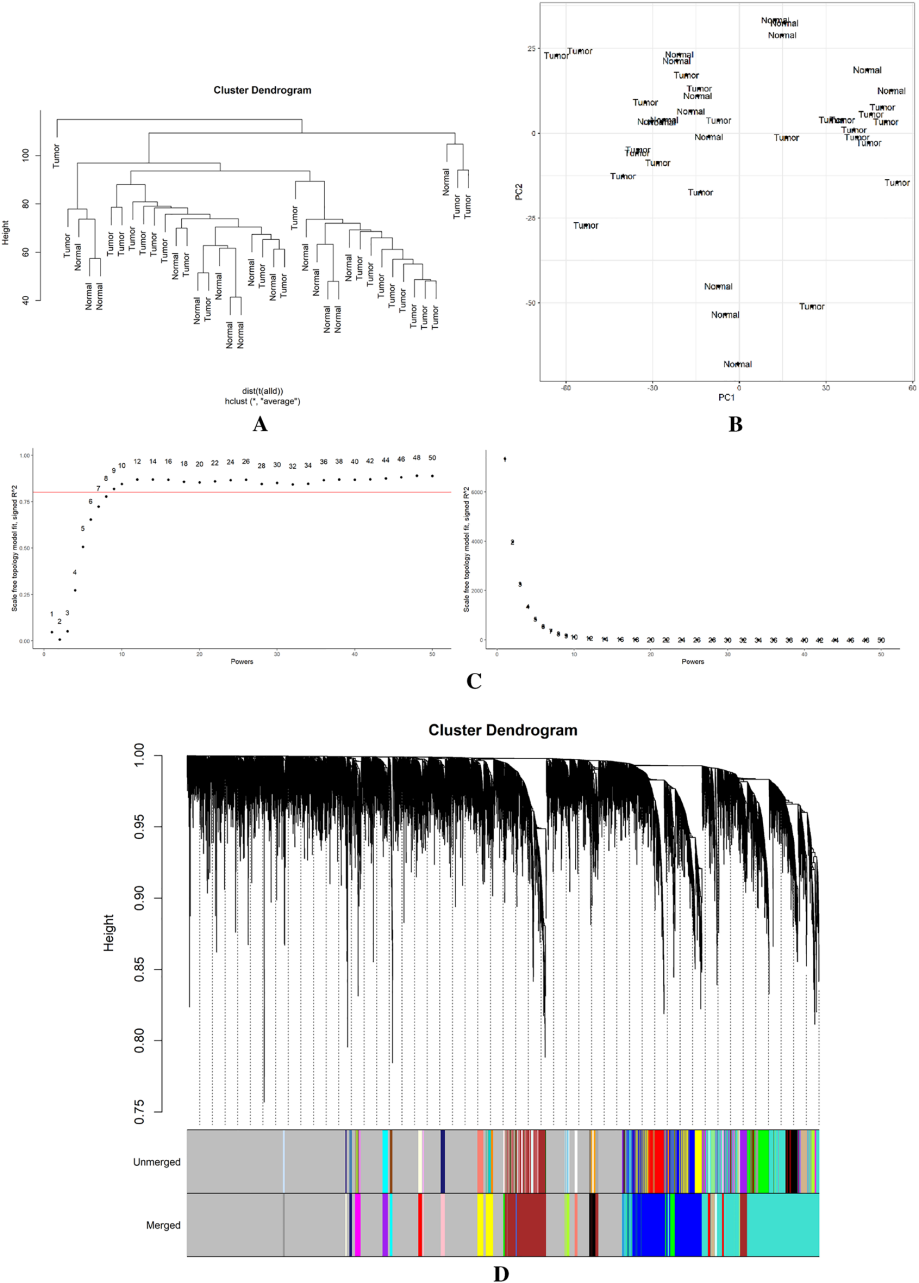

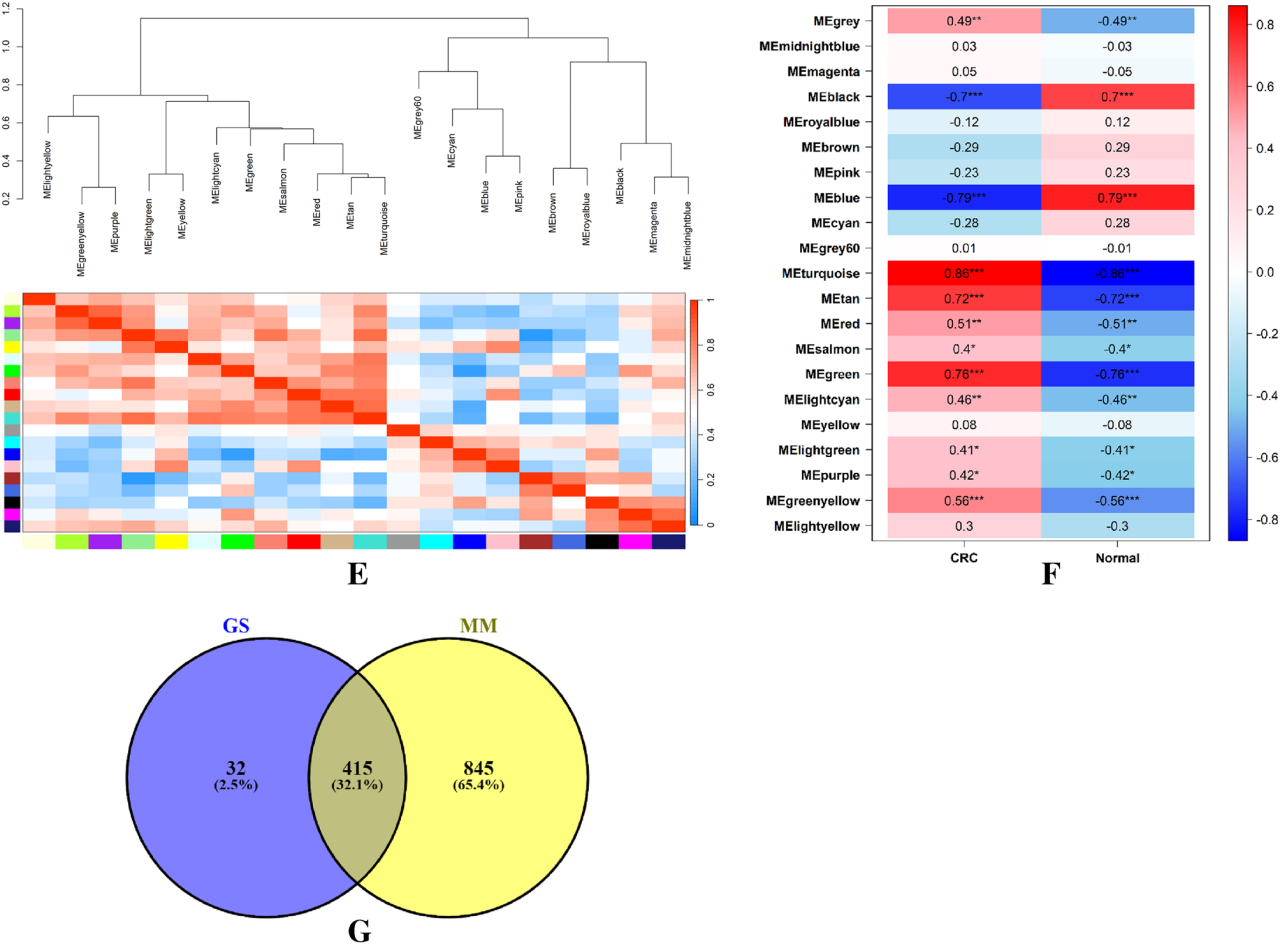
Weighted correlation network analysis. (**A**) Hierarchical clustering of samples to detect the outlier samples. (**B**) Principal component analysis to detect the outlier samples. (**C**) Scale independence (left) and mean connectivity (right). (**D**) Using a hierarchical clustering of genes based on the 1-TOM matrix, the co-expression network modules Cluster dendrogram is arranged in a certain order. Various modules are represented by various colors. (**E**) Hierarchical clustering of modules (above) and heatmap of trait and modules (below). (**F**) Module-trait relationships. Each column is a clinical feature (CRC and normal), and each row denotes a color module. The correlation coefficient is displayed in each cell. (**G**) intersection of GS and MM.

A total of 39 samples from merged dataset were used for WGCNA using WGCNA package in R. Then, we used principal component analysis and samples hierarchical clustering to eliminate outlier samples (Fig. 4A,B). We eliminated 4 samples because of clustering and carried on with the analysis using the remaining data. Soft threshold value for the dataset was chosen with a cutoff R2 value of 0.9. As the network follows the power law distribution at this value (Fig. 4C), it is more similar to the condition of a true biological network. In order to merge the similar modules, the minimum module size was set to 30 with a 025 height cut (Fig. 4D). Figure 4E depicts every gene co-expression module. Twenty co-expressed gene modules were identified, with gray modules denoting genes that were not co-expressed. Each module was given a color name. With 24 genes, the royalblue module is the smallest. The turquoise module, which has 2434 genes, is also the biggest module at the moment. In addition, the 7659 genes that are not associated with any module are represented by the background's grey color (Table 3). For the enrichment analysis, we selected modules with correlation coefficients of 0.7 or above (Fig. 4F). As a result, turquoise (0.86), tan (0.72) and green (0.76) modules were selected for further analysis. By evaluating the correlation between module eigengenes (MEs) and traits and the correlation between gene expression profiles and traits, respectively, we measured the membership module (MM) of these three modules and gene significance (GS) (Table S2). The similar genes GS and MM were lastly regarded as the most significant genes (Fig. 4G).

**Table 3:** Identified modules and their gene numbers.

Module color	Gene count
Grey	7659
Turquoise	2434
Blue	1456
Brown	1141
Yellow	301
Green	197
Red	187
Black	139
Pink	128
Magenta	127
Purple	121
Greenyellow	102
Tan	85
Salmon	64
Midnightblue	56
Cyan	56
Lightcyan	49
Grey60	42
Lightgreen	31
Lightyellow	30
Royalblue	24

### Key modules PPI network construction, identification of top clusters and hub genes

**Figure 5:**
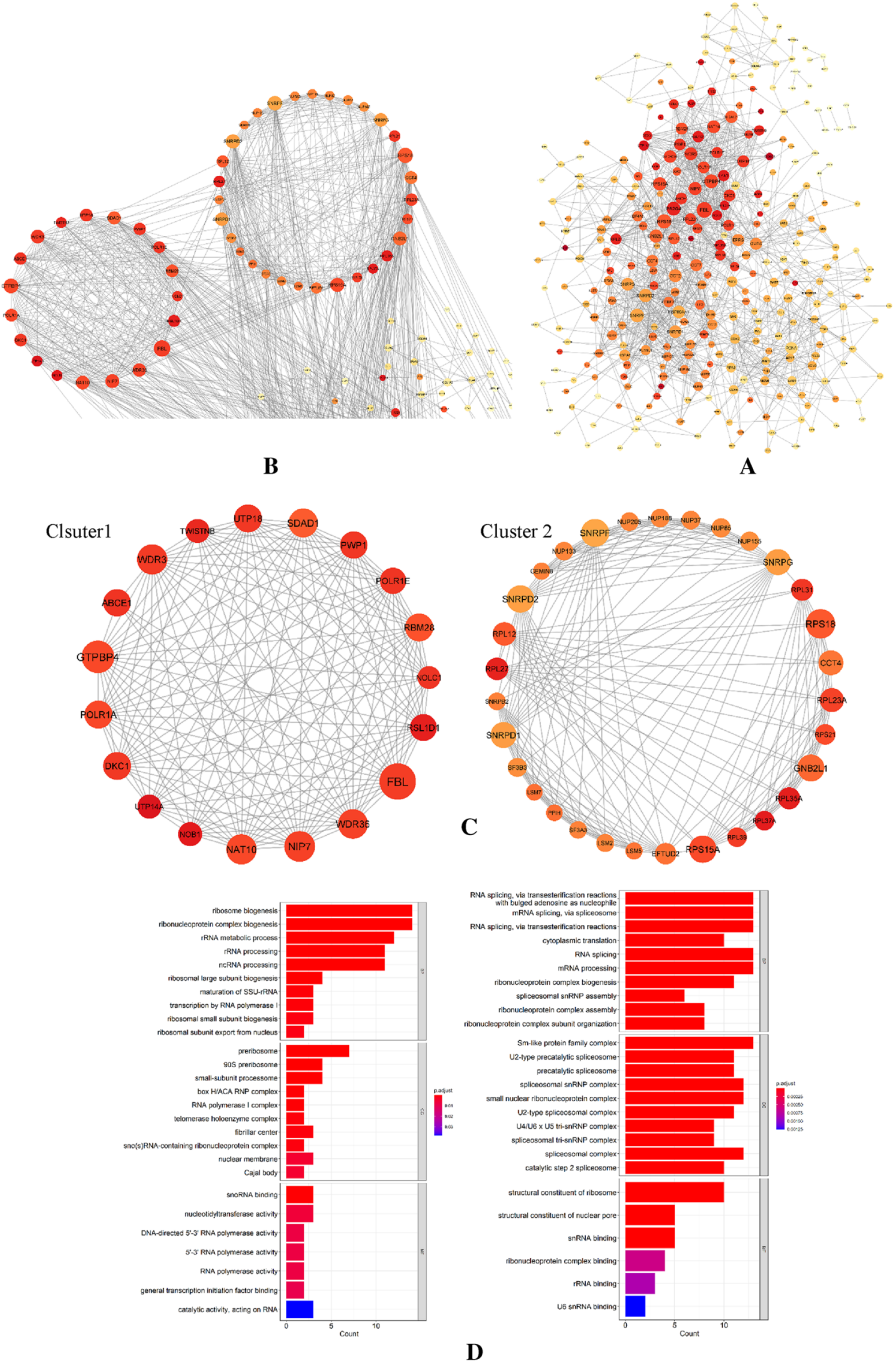

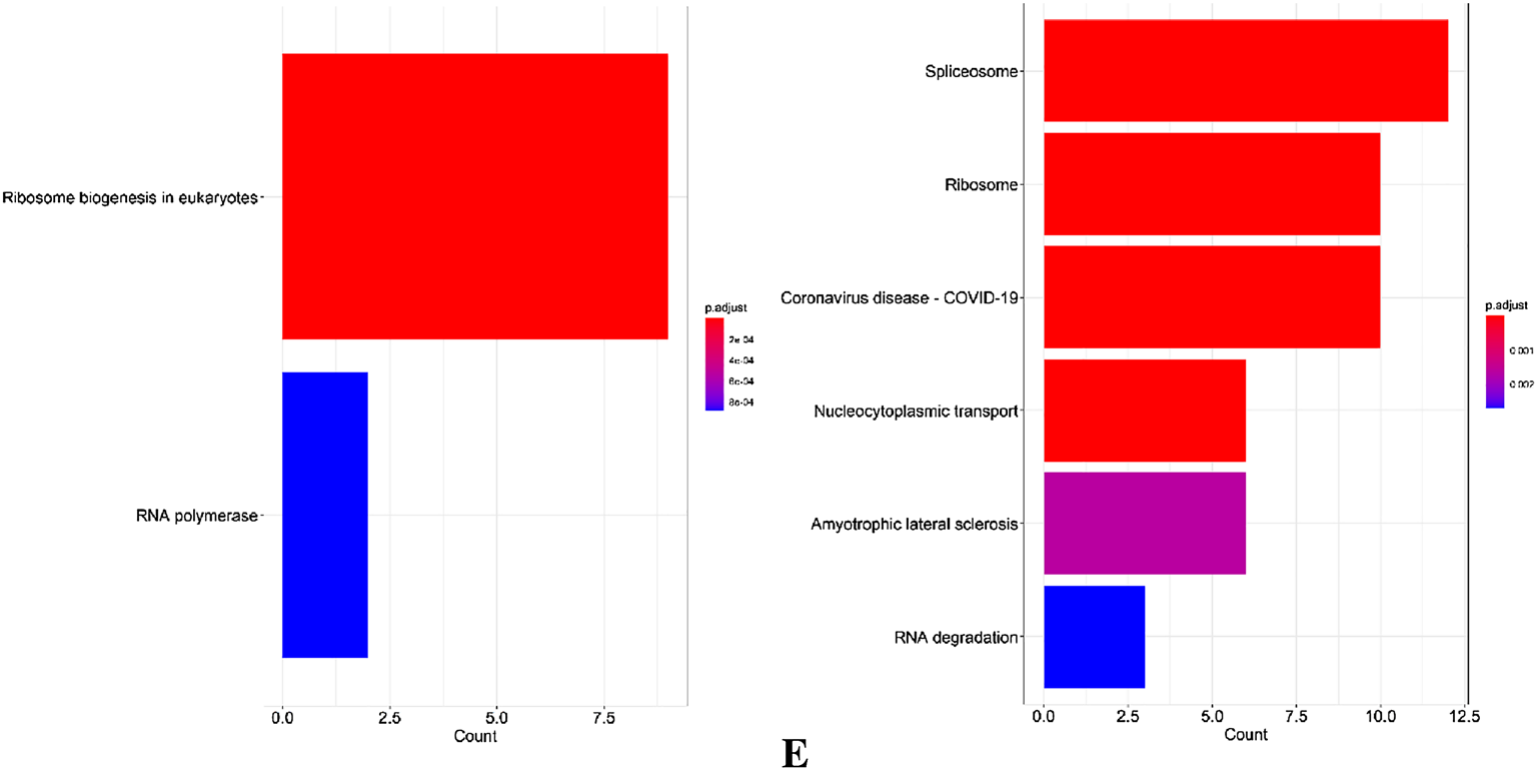
PPI network of most significant genes, two cluster modules extracted by MCODE and GO and KEGG pathway enrichment. (**A**) There were 294 nodes and 1416 edges in the protein–protein interaction network created by the most significant genes. A protein is represented by each node, and a protein–protein interaction is represented by each edge. Also, the size of the nodes corresponds to the degree centrality, while the color denotes the neighborhood connectivity centrality. (**B**) Display of clusters in the PPI network. (**C**) Two clusters extracted by MCODE. (**D**) GO enrichment of two clusters. (**E**) KEGG pathway enrichment of two clusters.

The PPI network of most significant genes (Fig. 5A) with 294 nodes and 1416 edges that was created from STRING was put into the MCODE plugin of Cytoscape 3.9 in order to identify the hub clusters (MCODE score > 10) (Fig. 5B). We identified two clusters (Fig. 5C). The first cluster with a score of 16,889 contained UTP18, SDAD1, PWP1, POLR1E, RBM28, RSL1D1, NOLC1, WDR36, NIP7, NAT10, NOB1, UTP14A, DKC1, POLR1A, GTPBP4, ABCE1, WDR3 and TWISTNB and the second cluster with a score of 12,267 contained NUP188, NUP37, NUP85, NUP155, SNRPG, RPL31, RPS18, CCT4, RPL23A, RPS21, GNB2L1, RPL35A, RPL37A, RPL39, RPS15A, EFTUD2, LSM2, LSM5, SF3A3, PPIH, LSM7, SF3B3, SNRPD1, SNRPB2, RPL27, RPL12, SNRPD2, GEMIN6, NUP133, SNRPF and NUP205. Next, we performed GO and KEGG enrichment analysis of two hub clusters using clusterProfiler package in R (Fig. 5D,E).

### PPI network hub genes detection

**Figure 6:**
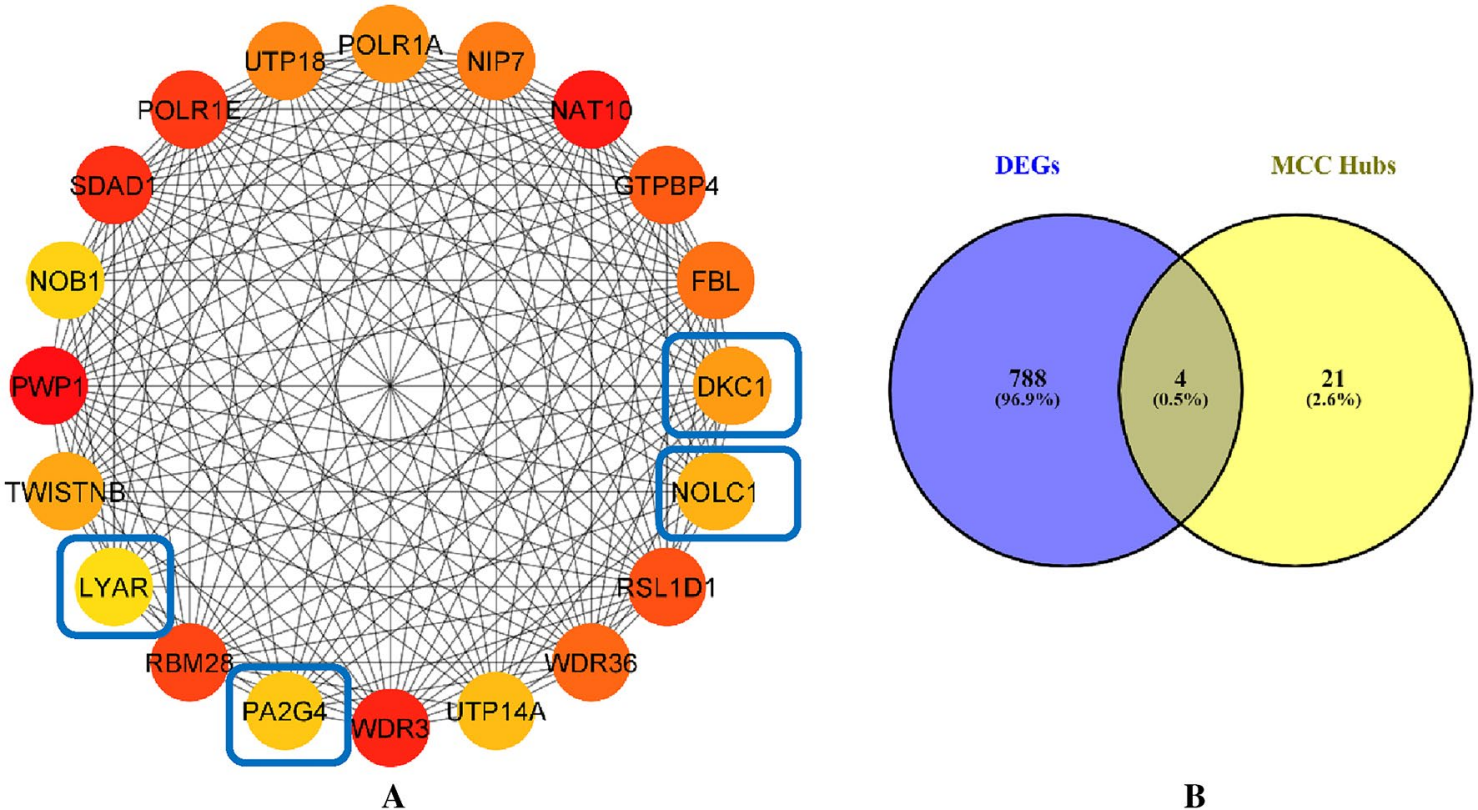
Obtaining the hub genes of PPI network using the maximal clique centrality algorithm in cytohubba plugin. (**A**) Top 25 hub genes with highest MCC. (**B**) Intersection between hub genes and DEmRNAs.

We identified hub genes of PPI network using cytohubba plugin. In this case, we selected 25 hub genes with highest maximal clique centrality (MCC) (Fig. 6A). Then, the intersection of genes between hub genes and DEmRNAs (Fig. 6B) showed that DKC1, PA2G4, LYAR and NOLC1 were the clinically final hub genes of CRC.

### Analyzing the regulatory networks of the TF-final hub genes and miRNAs

**Figure 7:**
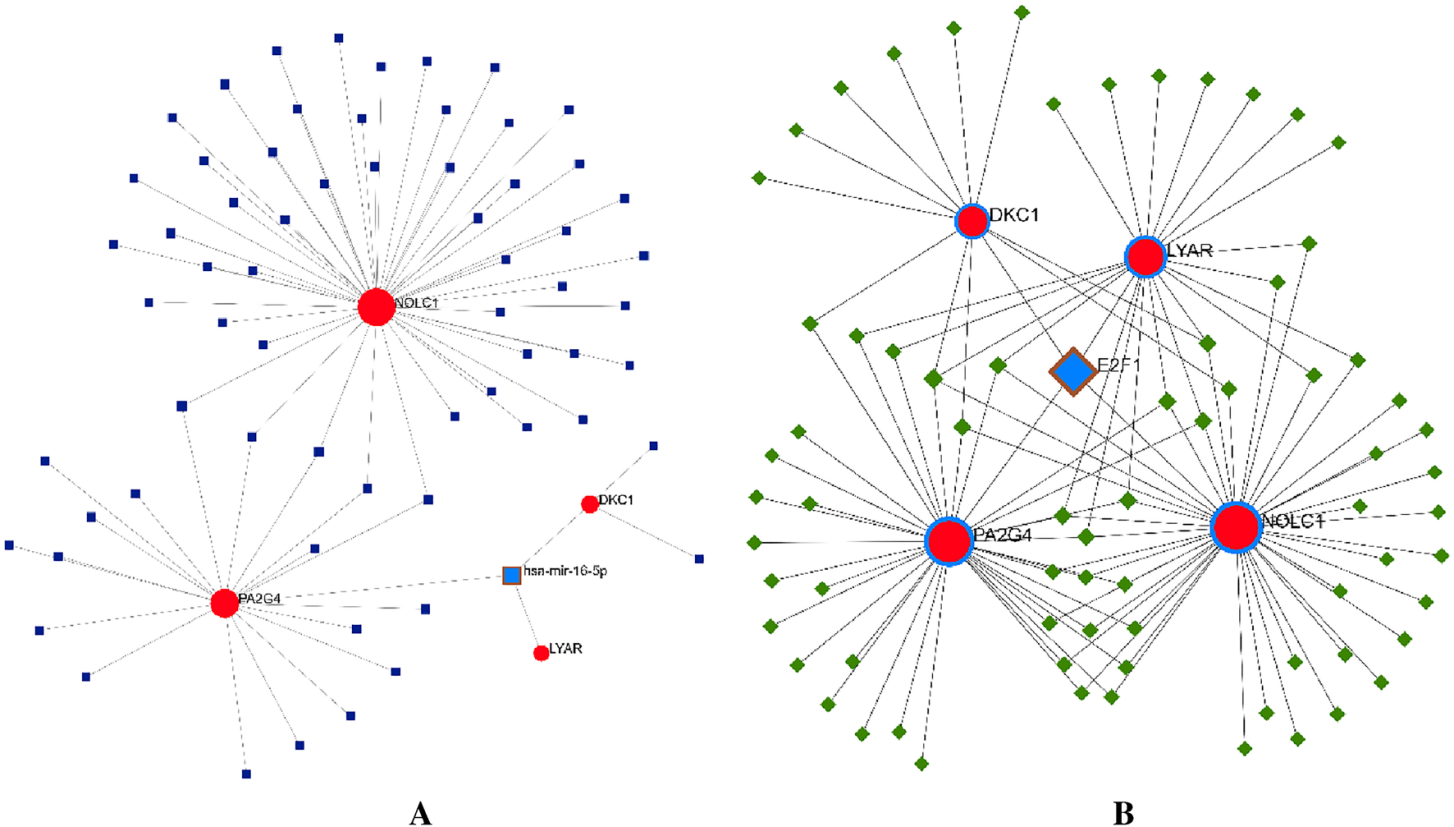
Construction of miRNA- and TF-final hub genes regulatory networks. (**A**) In the miRNA-final hub genes regulatory network, it is shown that has-miR-16-5p is related to the DKC1, LYAR and PA2G4. (**B**) In the TF-final hub genes regulatory network, it is shown that E2F1 is related to the DKC1, LYAR, PA2G4 and NOLC1.

There are several ways that miRNAs can control how genes are expressed. Networkanalyst online database was utilized to collect miRNAs that target hub genes (Fig. 7A), and in this case, we used the miRTarBase v8.0 database to discover hub miRNA interactions. Hsa-miR-16-5p was regarded as a key miRNA for the development of CRC because it interacted with the three final hub genes (degree 3). Also, we got TFs that target final hub genes from the NetworkAnalyst database (Fig. 7B), and we selected the ChEA database to find the TFs that do so. According to the TF-hub gene network, the E2F1 controls each of the four hub genes and may be important for the development of CRC.

### The genetic alterations of final hub genes in CRC patients

**Figure 8:**
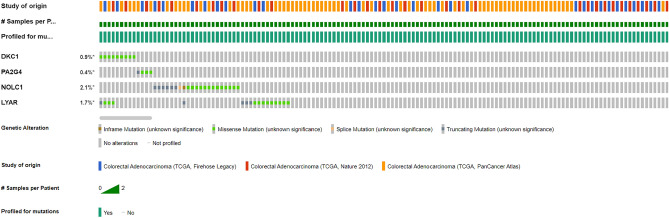
The genetic changes of the last hub genes in individuals with COAD. An asterisk (*) indicates that the gene has undergone mutations.

In colorectal adenocarcinoma TCGA datasets, we looked at the final hub genes mutations using the cBioPortal database. These datasets contained 1510 samples from 3 studies. As a result, out of a total of 981 samples, gene DKC1 was altered in 9 samples, PA2G4 in 4, NOLC1 in 21, and LYAR in 17 samples (Fig. 8). Also, it was discovered that the LYAR and DKC1 co-occur with mutations, with a q value of less than 0.001 (Table 4).

**Table 4:** Mutually exclusive mutation pattern between the final hub gene pairs.

A	B	Neither	A Not B	B Not A	Both	Log2 Odds Ratio	p value	q value	Tendency
**DKC1**	**LYAR**	**959**	**5**	**13**	**4**	** > 3**	** < 0.001**	** < 0.001**	**Co-occurrence**
NOLC1	LYAR	944	20	16	1	1.561	0.310	1.000	Co-occurrence
DKC1	NOLC1	951	9	21	0	< − 3	0.822	1.000	Mutual exclusivity
PA2G4	NOLC1	956	4	21	0	< − 3	0.917	1.000	Mutual exclusivity
PA2G4	LYAR	960	4	17	0	< − 3	0.932	1.000	Mutual exclusivity
DKC1	PA2G4	968	9	4	0	< − 3	0.964	1.000	Mutual exclusivity

### Validation of the expression of the final hub genes

**Figure 9:**
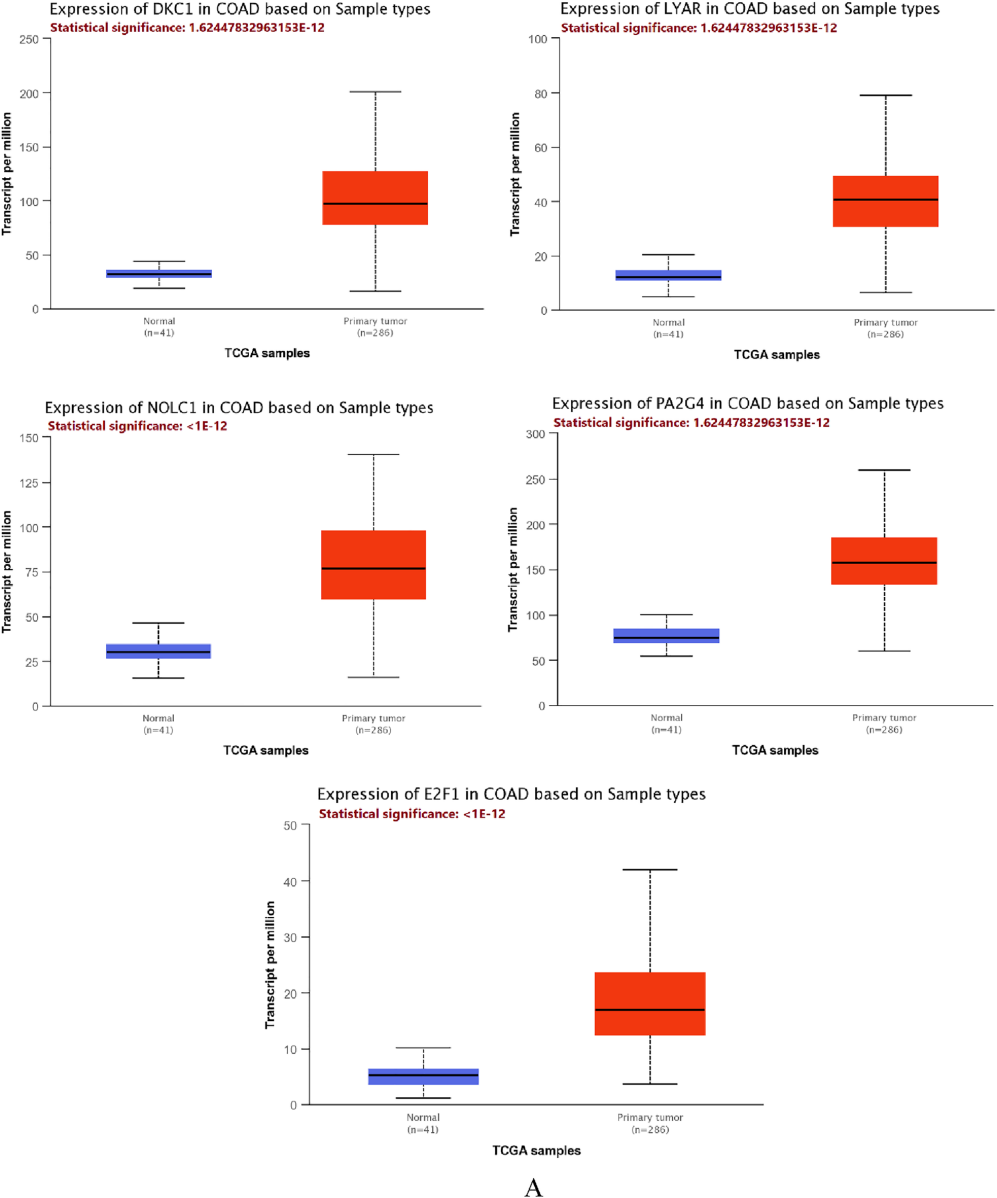

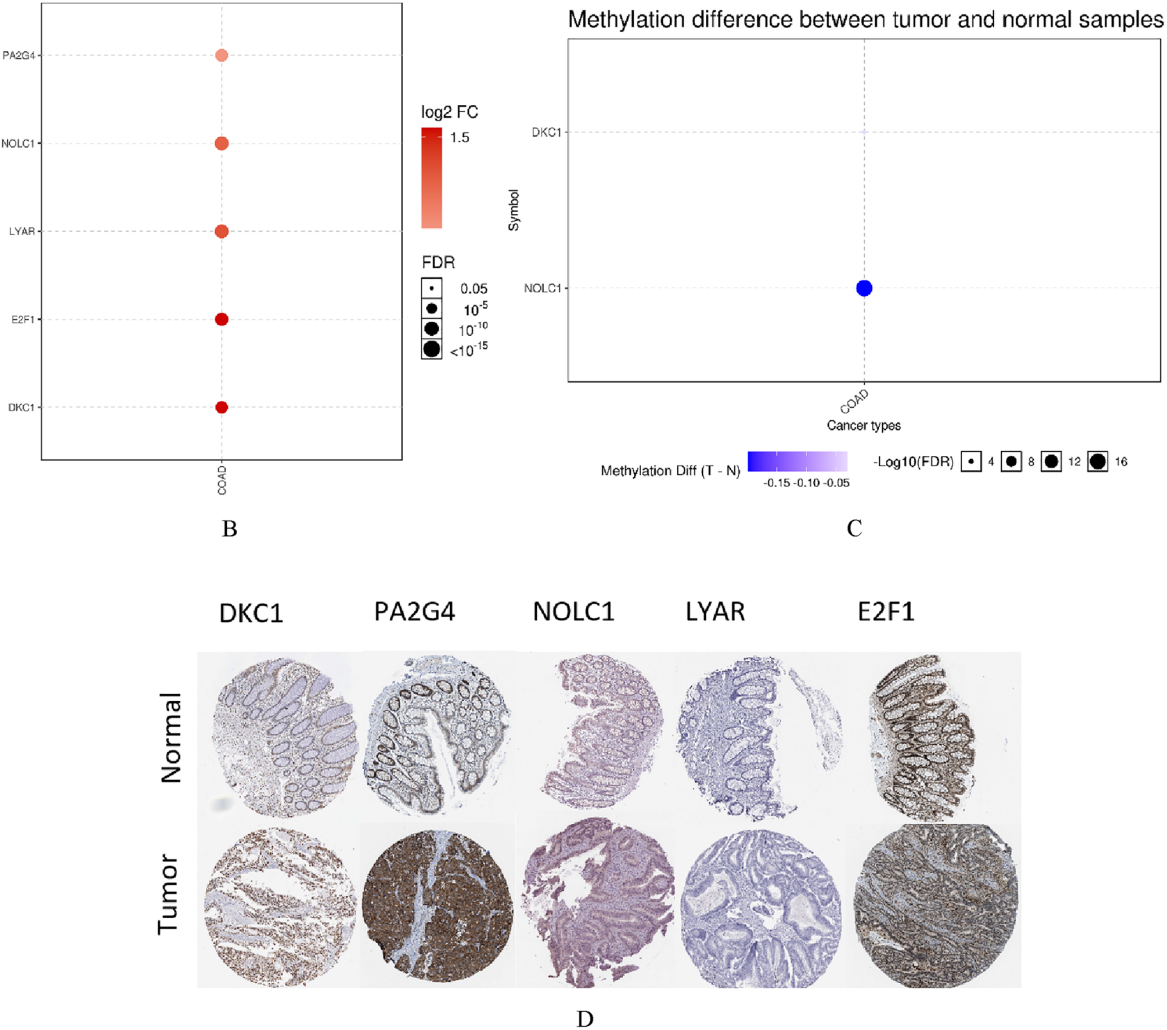
Validation of the final hub expression pattern in COAD. (**A**) DKC1, PA2G4, NOLC1, LYAR, and E2F1 expression patterns in COAD and normal samples were taken from the UALCAN database. (**B**) DKC1, PA2G4, NOLC1, LYAR, and E2F1 expression in COAD and normal samples from the GSCALite database. (**C**) Based on methylation difference between COAD and normal samples from the GSCALite database, there is a difference in the methylation of DKC1 and NOLC1; as the circle size increases, the level of significance becomes greater. Moreover, as the circle color shifts towards dark blue, it indicates a greater reduction in methylation in tumor samples. (**D**) Immunohistochemistry (IHC) of the DKC1 (DKC1 normal sample from patient 634; DKC1 COAD sample from patient 192), PA2G4 (PA2G4 normal sample from patient 1423; PA2G4 COAD sample from patient 2106), NOLC1 (NOLC1 normal sample from patient 1958; NOLC1 COAD sample from patient 4721), LYAR (LYAR normal sample from patient 2040; LYAR COAD sample from patient 4724), and E2F1 (E2F1 normal sample from patient 1423; E2F1 COAD sample from patient 2948) in COAD and normal samples from the HPA database.

We looked up the expression patterns of the final five hub genes (DKC1, PA2G4, NOLC1, LYAR, and E2F1) in several databases to check their reliability. According to the UALCAN database, the expression levels of every gene were noticeably greater in COAD than in normal samples (Fig. 9A; Table 5). According to the Gene Set Cancer Analysis Lite (GSCALite) database, all of these hub genes were also considerably enhanced in COAD (Fig. 9B). The methylation of these genes was also examined using the GSCALite database, and we discovered that NOLC1 was hypomethylated based on methylation difference between tumor and normal samples in COAD (Fig. 9C), which may be connected to the high level of this gene expression in COAD. The Human Protein Atlas (HPA) database also revealed that protein levels of these five genes considerably greater in tumor samples compared to normal samples (Fig. 9D).

**Table 5:** Statistical significance of the final hub genes in colorectal adenocarcinoma (COAD) TCGA data according on sample types.

Hub genes	Statistical significance of expression value
NOLC1	< 1E−12
E2F1	< 1E−12
LYAR	1.62447832963153E−12
PA2G4	1.62447832963153E−12
DKC1	1.62447832963153E−12

## Discussion

CRC is regarded as a cancer with high burden in the world needing urgent identification of appropriate diagnostic biomarkers. High throughput techniques are valuable tools for comparison of expression profiles of normal and cancerous cells to find biomarkers. WGCNA is a method for identification of interconnections between genes and recognition of differentially expressed genes between two sets of samples^25^. In the current study, we used this method and find final five hub genes, namely DKC1, PA2G4, NOLC1, LYAR, and E2F1. Expression levels of these gene were noticeably greater in COAD than in normal samples. Notably, NOLC1 was hypomethylated in COAD. Therefore, overexpression of this gene can be explained by the phenomenon.

Functionally, DKC1 has been shown to enhance angiogenesis through increasing HIF-1α transcription. Moreover, DKC1 can facilitate metastasis in CRC^26^. PA2G4 has also been shown to be highly expressed in a variety of cancers, including cervical cancer, CRC, nasopharyngeal carcinoma and salivary carcinoma^27–30^. In hepatocellular carcinoma, PA2G4 can promote the metastasis through increasing stability of FYN transcript in a YTHDF2-dependent manner^31^. NOLC1 is involved in determination of multidrug resistance phenotype in non-small cell lung cancer^32^. LYAR has been found to promote CRC cell mobility through activating galectin-1 expression^33^. Finally, E2F1 is a transcription factor that binds to DNA with dimerization partner proteins. This transcription factor has fundamental roles in the development of CRC^34^.

Notably, these genes have been found to be mutated in CRC samples. Thus, several lines of evidence show fundamental roles of DKC1, PA2G4, NOLC1, LYAR, and E2F1 in CRC.

To sum up, the bioinformatics strategy used in the current study revealed important roles of DKC1, PA2G4, NOLC1, LYAR, and E2F1 in the CRC carcinogenesis and potentiates these genes as biomarkers for detection of CRC and therapeutic targets for this cancer.

### Supplementary Information

Supplementary Information.

## Data Availability

The datasets used and/or analyzed during the current study are available as GSE141174 (https://www.ncbi.nlm.nih.gov/geo/query/acc.cgi?acc=GSE141174), GSE184093 (https://www.ncbi.nlm.nih.gov/geo/query/acc.cgi?acc=GSE184093) and GSE206800 (https://www.ncbi.nlm.nih.gov/geo/query/acc.cgi?acc=GSE206800).

## References

[1] Cheng L, Eng C, Nieman LZ, Kapadia AS, Du XL (2011). Trends in colorectal cancer incidence by anatomic site and disease stage in the United States from 1976 to 2005. Am. J. Clin. Oncol..

[2] Xiao Y, Li T, Xue Q, Miao L (2020). Long non-coding RNA GHET1/miR-105/RAP2B axis regulates the progression of acute myeloid leukemia. J. Cancer.

[3] Duan B, Morgado-Diaz JA (2022). Gastrointestinal Cancers.

[4] Inadomi JM (2012). Adherence to colorectal cancer screening: A randomized clinical trial of competing strategies. Arch. Intern. Med..

[5] Langfelder P, Horvath S (2008). WGCNA: An R package for weighted correlation network analysis. BMC Bioinform..

[6] Ghafouri-Fard S, Hussen BM, Badrlou E, Abak A, Taheri M (2021). MicroRNAs as important contributors in the pathogenesis of colorectal cancer. Biomed. Pharmacother..

[7] Ghafouri-Fard S (2021). Function of circular RNAs in the pathogenesis of colorectal cancer. Biomed. Pharmacother..

[8] Ghafouri-Fard S, Hussen BM, Gharebaghi A, Eghtedarian R, Taheri M (2021). LncRNA signature in colorectal cancer. Pathol. Res. Pract..

[9] Guo C, Xie B, Liu Q (2022). Weighted gene co-expression network analysis combined with machine learning validation to identify key hub biomarkers in colorectal cancer. Funct. Integr. Genom..

[10] Cao L (2023). Development and validation of an RBP gene signature for prognosis prediction in colorectal cancer based on WGCNA. Hereditas.

[11] Lin L (2022). Construction of a co-expression network and prediction of metastasis markers in colorectal cancer patients with liver metastasis. J. Gastrointest. Oncol..

[12] Leek JT, Johnson WE, Parker HS, Jaffe AE, Storey JD (2012). The sva package for removing batch effects and other unwanted variation in high-throughput experiments. Bioinformatics.

[13] Bader GD, Hogue CWV (2003). An automated method for finding molecular complexes in large protein interaction networks. BMC Bioinform..

[14] Kanehisa M, Goto S (2000). KEGG: Kyoto encyclopedia of genes and genomes. Nucleic Acids Res..

[15] Kanehisa M, Furumichi M, Sato Y, Kawashima M, Ishiguro-Watanabe M (2023). KEGG for taxonomy-based analysis of pathways and genomes. Nucleic Acids Res..

[16] Kanehisa M (2019). Toward understanding the origin and evolution of cellular organisms. Protein Sci..

[17] Wu T (2021). clusterProfiler 40: A universal enrichment tool for interpreting omics data. Innovation.

[18] Chin C-H (2014). cytoHubba: Identifying hub objects and sub-networks from complex interactome. BMC Syst. Biol..

[19] Zhou G (2019). NetworkAnalyst 3.0: A visual analytics platform for comprehensive gene expression profiling and meta-analysis. Nucleic Acids Res..

[20] Cerami E (2012). The cBio cancer genomics portal: An open platform for exploring multidimensional cancer genomics data. Cancer Discov..

[21] Chandrashekar DS (2017). UALCAN: A portal for facilitating tumor subgroup gene expression and survival analyses. Neoplasia.

[22] Liu CJ (2018). GSCALite: A web server for gene set cancer analysis. Bioinformatics.

[23] Uhlén M (2015). Proteomics. Tissue-based map of the human proteome. Science.

[24] Ritchie ME (2015). limma powers differential expression analyses for RNA-sequencing and microarray studies. Nucleic Acids Res..

[25] Qiu X (2020). Weighted gene co-expression network analysis identified MYL9 and CNN1 are associated with recurrence in colorectal cancer. J. Cancer.

[26] Hou P (2020). DKC1 enhances angiogenesis by promoting HIF-1α transcription and facilitates metastasis in colorectal cancer. Br. J. Cancer.

[27] Liu H, Li Z, Li L, Peng H, Zhang Z (2015). EBP1 suppresses growth, migration, and invasion of thyroid cancer cells through upregulating RASAL expression. Tumor Biol..

[28] Liu L, Xu D, Yang S, Li X (2015). Ebp1 protein expression in cervical cancer tissue and its significance. Genet. Mol. Res..

[29] Xu Y, Cai H, Tu W, Ding L, Luo R (2021). Increased PA2G4 expression is an unfavorable factor in nasopharyngeal carcinoma. Appl. Immunohistochem. Mol. Morphol..

[30] Sun J (2012). Expression of ERBB3 binding protein 1 (EBP1) in salivary adenoid cystic carcinoma and its clinicopathological relevance. BMC Cancer.

[31] Sun S (2022). PA2G4 promotes the metastasis of hepatocellular carcinoma by stabilizing FYN mRNA in a YTHDF2-dependent manner. Cell Biosci..

[32] Huang H (2018). Identification and validation of NOLC1 as a potential target for enhancing sensitivity in multidrug resistant non-small cell lung cancer cells. Cell. Mol. Biol. Lett..

[33] Wu Y (2015). LYAR promotes colorectal cancer cell mobility by activating galectin-1 expression. Oncotarget.

[34] Fang Z, Lin M, Li C, Liu H, Gong C (2020). A comprehensive review of the roles of E2F1 in colon cancer. Am. J. Cancer Res..

