# Supplementary material for: Identification of diagnostic biomarkers via weighted correlation network analysis in colorectal cancer using a system biology approach
Source: Sci Rep. 2023 Aug 21;13:13637. doi: 10.1038/s41598-023-40953-5 (PMC10442394; doi:10.1038/s41598-023-40953-5)
Supplement: Supplementary file 1 — Supplementary Information. [file 41598_2023_40953_MOESM1_ESM.docx]

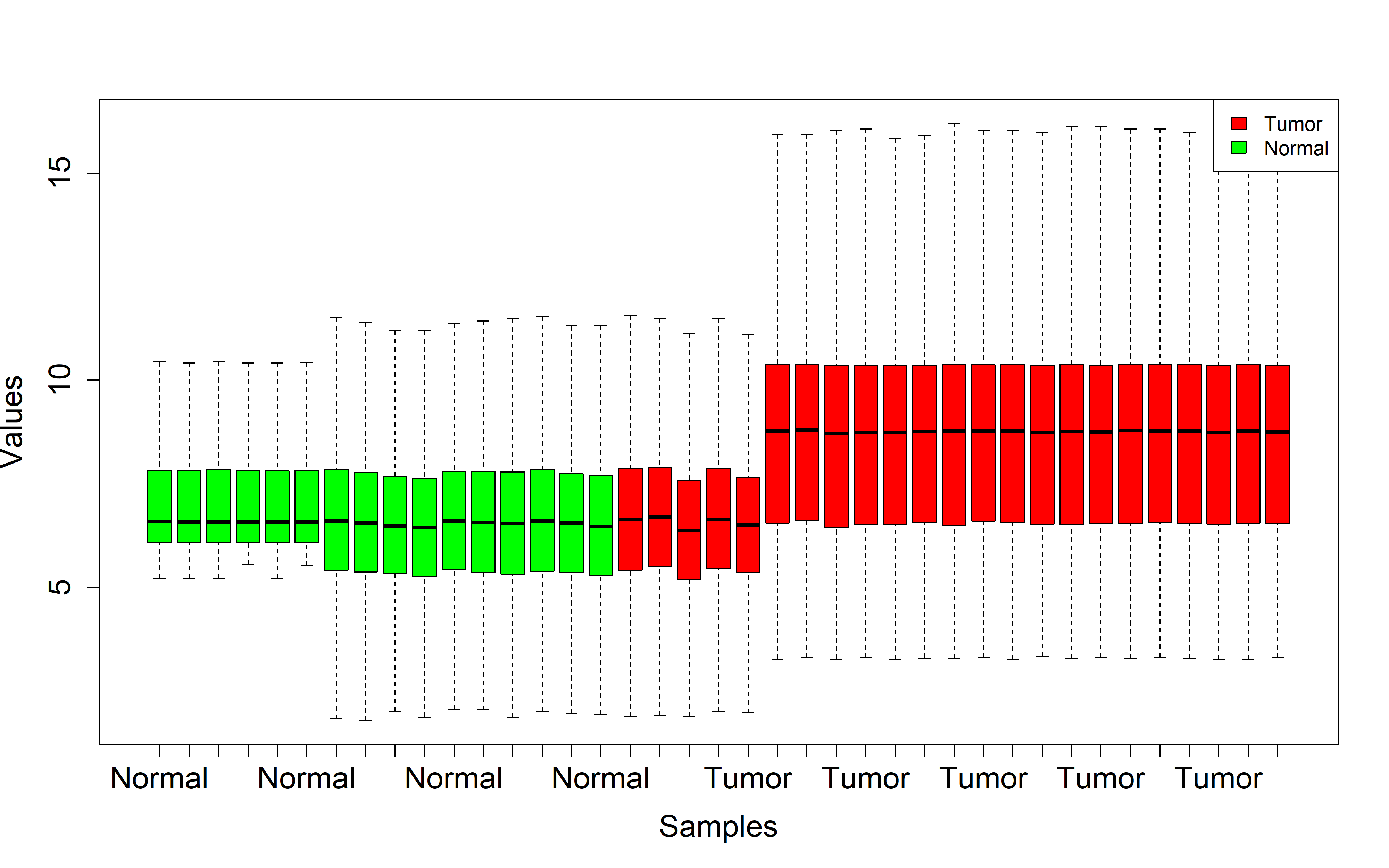

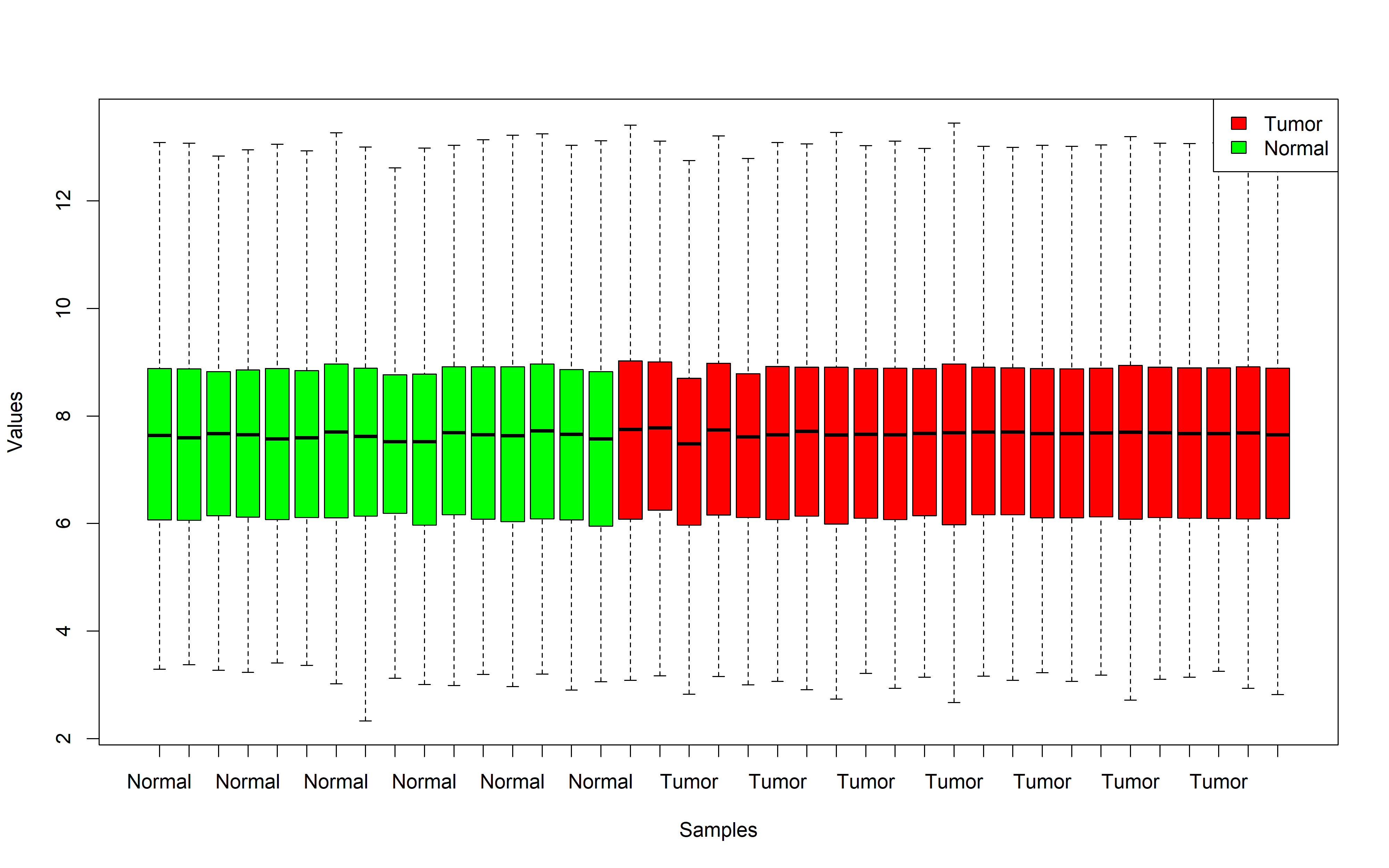


Figure S1. Boxplots for the raw data and normalized data after batch effect removal. CRC samples are indicated by red boxes, whereas normal samples are shown by green boxes.


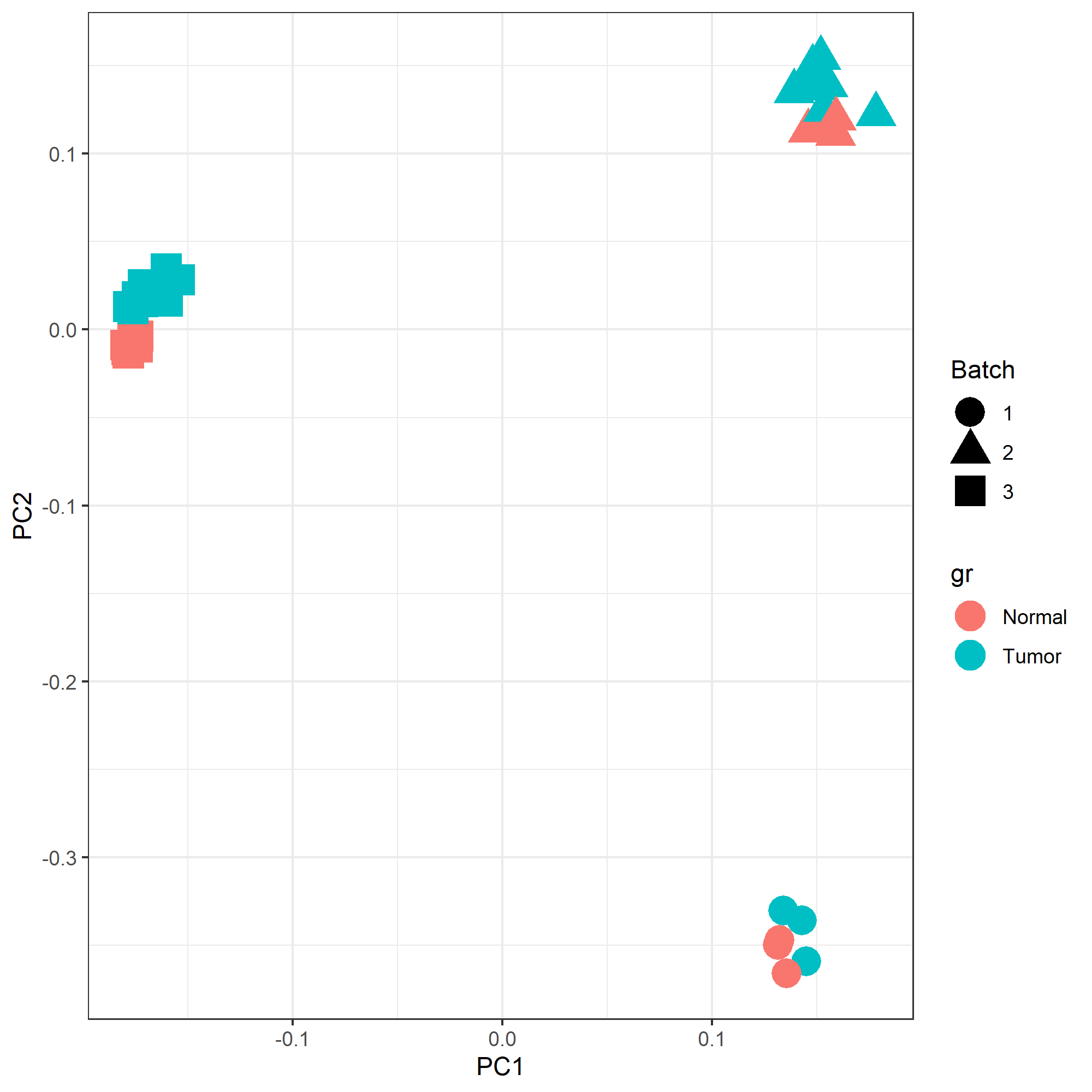


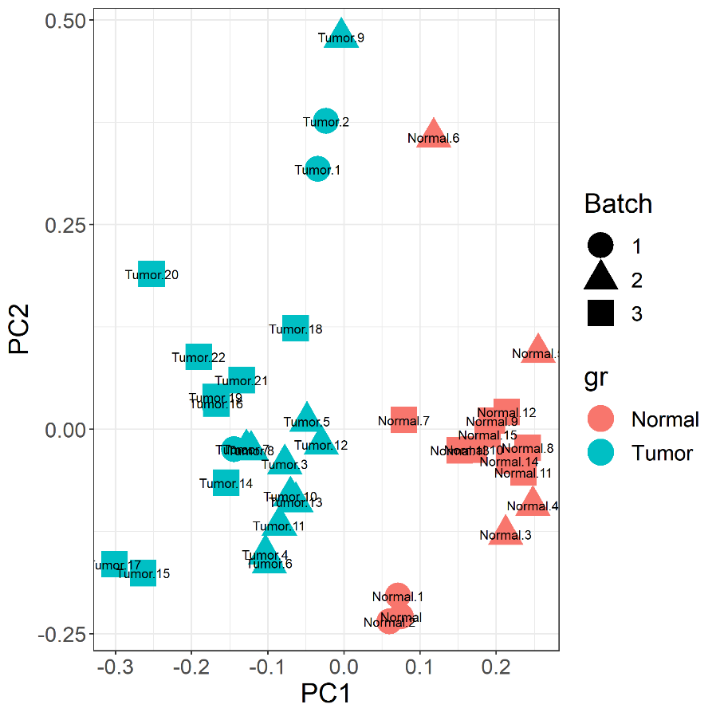


Figure S2. Principal component analysis plot. PCA plot of merged data without batch effect removal and with batch effect removal using ComBat.

Table S1. Differentially expressed mRNAs.

**Downregulated DEmRNAs**

| Symbol | Adjusted P.value | Log2FC |
| --- | --- | --- |
| CA1 | 1.93031129373484e-13 | -5.43200382817883 |
| TMIGD1 | 1.93031129373484e-13 | -4.71090079551194 |
| GUCA2A | 6.11456410108153e-12 | -3.9818014586975 |
| SLC4A4 | 1.38388586917794e-11 | -3.79749389459274 |
| AQP8 | 2.7357728781742e-11 | -4.1942055330619 |
| SLC26A2 | 4.70786688665038e-11 | -3.00031717713978 |
| SLC17A4 | 4.70786688665038e-11 | -2.86200874740963 |
| ABCG2 | 7.96863856678851e-11 | -2.74867826972307 |
| OTOP2 | 9.62057963226759e-11 | -2.36580014337601 |
| ABCA8 | 1.52810792627338e-10 | -3.09751241295377 |
| PRDX6 | 2.3611937219216e-10 | -1.12852038964106 |
| CA7 | 3.63048383328415e-10 | -2.88902122774958 |
| CA4 | 3.76734620716036e-10 | -3.54261265571536 |
| ENTPD5 | 3.76734620716036e-10 | -1.77797839196479 |
| SLC30A10 | 4.65635365090732e-10 | -3.16998616823706 |
| PLP1 | 4.65635365090732e-10 | -1.44243624599403 |
| CLCA4 | 6.74412679226954e-10 | -4.73611199689056 |
| ADH1C | 9.56795372567258e-10 | -2.76000410575014 |
| TTLL6 | 1.05404329972473e-09 | -1.69785191883478 |
| CDKN2B | 1.07184057866666e-09 | -1.42519032943533 |
| HSD11B2 | 1.29506303516431e-09 | -2.31781995895581 |
| PRKG2 | 1.38608925685943e-09 | -2.20505432585286 |
| TRPM6 | 1.54957858083036e-09 | -2.70176156925826 |
| SCNN1B | 1.95717224518614e-09 | -2.46113669315285 |
| GUCA2B | 1.95717224518614e-09 | -2.65327161011568 |
| ACAA1 | 2.12701949865053e-09 | -1.01331102924863 |
| ASPA | 2.12701949865053e-09 | -2.60656488939639 |
| PKIB | 2.41278727284026e-09 | -2.58866008632448 |
| SULT1A2 | 2.64133741843165e-09 | -1.28743858940613 |
| CA2 | 2.64133741843165e-09 | -3.00698745469448 |
| ZG16 | 2.64133741843165e-09 | -4.36657347158967 |
| CLDN23 | 2.70640773837102e-09 | -1.8357385501517 |
| PCK1 | 3.11535078802171e-09 | -2.33608145300689 |
| USP2 | 3.29146668995439e-09 | -1.97125305278704 |
| BMP3 | 5.48566830730311e-09 | -2.50737759394706 |
| SYTL2 | 5.48566830730311e-09 | -1.35410059183212 |
| SLC26A3 | 5.48566830730311e-09 | -4.32984618703767 |
| BEST4 | 6.87235020353782e-09 | -1.83544404199277 |
| POLD4 | 7.07067726151624e-09 | -1.01303566921543 |
| CHAD | 7.07067726151624e-09 | -1.19140993324945 |
| VSIG2 | 7.41873486113775e-09 | -3.28265482741159 |
| PLCD1 | 7.82991734895517e-09 | -1.47094844289536 |
| PDE9A | 7.99944674784065e-09 | -2.13854544376846 |
| NR5A2 | 8.90098513063504e-09 | -1.55467880508671 |
| HSD17B2 | 9.00568324132585e-09 | -2.53477116620236 |
| CLCA1 | 9.14732736571730e-09 | -3.80818517865493 |
| PLCE1 | 9.42186758597276e-09 | -2.00963988647383 |
| SEMA6A | 9.62381732369383e-09 | -1.97323695441489 |
| PEX26 | 9.62381732369383e-09 | -1.11338535270057 |
| MS4A12 | 9.73008672291592e-09 | -4.3290978576324 |
| UGT2A3 | 1.05133655154321e-08 | -2.96865772000624 |
| ZZEF1 | 1.08396189904563e-08 | -1.33538322147825 |
| CD177 | 1.2672132318525e-08 | -2.65926029567328 |
| SLC25A34 | 1.32563012934752e-08 | -1.2737405668842 |
| MYOT | 1.56340777861956e-08 | -1.48237462388562 |
| GPT | 1.57030810356207e-08 | -2.05985165034849 |
| NR3C2 | 1.79256877913293e-08 | -1.97303134429694 |
| ANPEP | 1.81969315189323e-08 | -3.59652136262575 |
| VIPR1 | 1.93657510414907e-08 | -1.55128213257126 |
| PDE6A | 1.93657510414907e-08 | -1.72188027024148 |
| C1orf115 | 2.04361286327045e-08 | -1.63457394201219 |
| PDZD3 | 2.70072315083972e-08 | -2.00703457176059 |
| MICALCL | 2.73798248204447e-08 | -1.17752457350005 |
| APPL2 | 2.88218657973344e-08 | -1.39120095284292 |
| KRT20 | 3.08930279845263e-08 | -2.59846770684588 |
| BCAS1 | 3.586626291184e-08 | -2.42377926212192 |
| LGI1 | 3.65049933800355e-08 | -1.62157454169569 |
| ABCA5 | 4.19051528055256e-08 | -1.23423099775935 |
| NEUROD1 | 4.43826588830206e-08 | -1.45640594373496 |
| ENTPD8 | 4.534312875583e-08 | -1.39224819448777 |
| PTPRH | 4.65997368079022e-08 | -1.62040474040692 |
| STMN2 | 4.89537499231906e-08 | -2.2343051466077 |
| PLAC8 | 4.95275884700547e-08 | -1.85375802940541 |
| SMPDL3A | 5.99092725460218e-08 | -1.89483428270984 |
| ACAA2 | 8.16978395721635e-08 | -1.2819194812797 |
| RNF152 | 9.0648296992515e-08 | -1.6865866521039 |
| TP53INP2 | 9.44878772008024e-08 | -1.47009176281308 |
| B3GALT4 | 1.02314408304332e-07 | -1.14795286764065 |
| MUSK | 1.14954932423051e-07 | -1.01338399618444 |
| BEST2 | 1.15383184881944e-07 | -1.82440829924133 |
| PLA2G10 | 1.20173277509396e-07 | -1.88541091735635 |
| CNTN3 | 1.2178492658999e-07 | -1.72687401518873 |
| TP53I3 | 1.2789902124459e-07 | -1.07214646710071 |
| LRRN2 | 1.28351230526341e-07 | -1.12460637789745 |
| SLC4A10 | 1.28684706598764e-07 | -1.69687404713522 |
| CNNM2 | 1.43900306101571e-07 | -1.1446578579021 |
| RILP | 1.45110649968548e-07 | -1.06758002129729 |
| ABHD3 | 1.47787017120586e-07 | -1.27161372897953 |
| TMCC3 | 1.49117390895834e-07 | -1.63621377922459 |
| GRAMD3 | 1.56751130625361e-07 | -1.19880911743456 |
| MARVELD3 | 1.8253780153893e-07 | -1.22077958034361 |
| ARRDC4 | 1.88389839738397e-07 | -1.10593772455118 |
| KLF4 | 1.94868775662989e-07 | -1.54640664815304 |
| TSPAN7 | 2.2766291920429e-07 | -1.90510459484321 |
| ATP8B1 | 2.30413513653552e-07 | -1.07121388137615 |
| TPK1 | 2.40751973087548e-07 | -1.13029588209031 |
| SLC35D1 | 2.43530447162156e-07 | -1.09904788567763 |
| METTL7A | 2.48456619037264e-07 | -1.43926115582403 |
| ALPI | 2.61723194619216e-07 | -1.30653688283001 |
| CAPN5 | 2.81816747950068e-07 | -1.37521390094257 |
| BMP2 | 2.82658141763953e-07 | -1.37476607389231 |
| GDPD2 | 2.85374743801639e-07 | -2.07604358755701 |
| CKB | 2.85374743801639e-07 | -1.97341801568051 |
| MIER3 | 2.87567557719211e-07 | -1.28741853311639 |
| GCNT2 | 2.87567557719211e-07 | -1.40362456879589 |
| CDC14A | 2.87567557719211e-07 | -1.28628048699089 |
| FMO5 | 2.8894926767093e-07 | -2.08099027178763 |
| AQP7 | 2.96788091488691e-07 | -1.04493126683025 |
| IQGAP2 | 2.98194590738442e-07 | -1.10473081376688 |
| GLP2R | 3.28568065716271e-07 | -1.3635902265931 |
| PRSS12 | 3.32787143137034e-07 | -1.23496205165487 |
| CCDC68 | 3.36383763457672e-07 | -1.72558103310581 |
| MEP1A | 3.36870792758197e-07 | -2.82047351885282 |
| NEDD4L | 3.54696685421042e-07 | -1.27453401874089 |
| SEMA6D | 3.66549343390878e-07 | -1.54393142857576 |
| EPHX2 | 3.83506619326866e-07 | -1.09061607028856 |
| TUBAL3 | 3.93996587639212e-07 | -1.52382942683406 |
| PIGZ | 4.31524229601452e-07 | -1.69342312095223 |
| CLDN8 | 4.32430902290589e-07 | -2.43657974201136 |
| SLCO2A1 | 4.35478103968253e-07 | -1.65646799994349 |
| CEACAM7 | 4.38908799468433e-07 | -3.32337328829199 |
| UGP2 | 4.51770898206217e-07 | -1.18642923458067 |
| ST6GALNAC6 | 4.67706608239331e-07 | -1.74193529002232 |
| SDCBP2 | 4.93462720538369e-07 | -1.9676135063306 |
| CADM2 | 5.02748207093985e-07 | -1.53665131633654 |
| ETFDH | 5.16582278454962e-07 | -1.20923768432922 |
| C15orf48 | 5.45022609780101e-07 | -1.79932839947281 |
| ADHFE1 | 5.52286485871272e-07 | -1.93814367111861 |
| ACACB | 5.63855862581458e-07 | -1.18601313801308 |
| SULT1B1 | 5.97366157278351e-07 | -1.86437730181965 |
| PLSCR4 | 6.22195790794973e-07 | -1.08847891402659 |
| MT1E | 6.74679678991869e-07 | -1.56437974514808 |
| GBA3 | 6.83198812004062e-07 | -1.9731038107398 |
| SLC39A5 | 6.86985752687367e-07 | -1.43419889266236 |
| PLD1 | 7.0566542482697e-07 | -1.08403600943826 |
| MMP28 | 7.35633227549107e-07 | -1.36736482029093 |
| BTNL3 | 7.66138984561104e-07 | -2.02257940039118 |
| SCIN | 7.71412809673428e-07 | -1.92851239267886 |
| PPARGC1A | 7.83267950839604e-07 | -2.25714539307808 |
| HHLA2 | 8.13955383340949e-07 | -2.34908366309772 |
| ZNF536 | 8.43477529120346e-07 | -1.06930940154806 |
| CNNM4 | 8.56129783469857e-07 | -1.14461785551791 |
| NAALADL1 | 8.65872844150144e-07 | -1.38854027797745 |
| SMPD1 | 8.96703105634e-07 | -1.03772271508295 |
| SLC22A18AS | 9.67196035465244e-07 | -1.33443929744109 |
| B4GALNT2 | 1.0178910195374e-06 | -3.15457100759272 |
| DHRS9 | 1.05628568407666e-06 | -2.38828856009334 |
| PDCD4 | 1.05665572121815e-06 | -1.13201427228663 |
| ARL14 | 1.10915110108731e-06 | -1.67304437467391 |
| TCF21 | 1.17655516013572e-06 | -1.33279849477695 |
| FRMD3 | 1.17671257750926e-06 | -1.44405783808212 |
| GFRA1 | 1.2033226375202e-06 | -1.27208977544886 |
| PLEKHH2 | 1.20523752496545e-06 | -1.30751901587939 |
| TMEM171 | 1.21323378747205e-06 | -1.96472288137974 |
| ST6GALNAC1 | 1.26737589545812e-06 | -1.86525183123247 |
| TMEM37 | 1.2812630192221e-06 | -1.24218051453912 |
| C5orf30 | 1.30191214371358e-06 | -1.06390395957793 |
| TCEA3 | 1.34379082136846e-06 | -1.45723185764447 |
| CNTN1 | 1.38289164205274e-06 | -1.44814073570964 |
| SMPD3 | 1.4912691382917e-06 | -1.17151560889278 |
| RXRG | 1.49432939129351e-06 | -1.31773993331147 |
| CDH19 | 1.61046364456018e-06 | -1.65154031227328 |
| TPH1 | 1.6386722348482e-06 | -2.67114630041392 |
| KRT12 | 1.6386722348482e-06 | -1.31985864934589 |
| AMPD1 | 1.66889515865302e-06 | -1.88632328684387 |
| CADM3 | 1.83049619008889e-06 | -1.4080106089611 |
| SCNN1G | 1.88195198224554e-06 | -2.03615942119413 |
| SST | 1.91239746018916e-06 | -1.99805926507431 |
| GSN | 1.96268447404654e-06 | -1.03540607395613 |
| EDN3 | 1.97636355558031e-06 | -1.8055902184425 |
| SLC30A4 | 1.97943093620323e-06 | -1.04183604366799 |
| LDHD | 1.99590951099464e-06 | -1.33456025809353 |
| HPSE2 | 2.08577233273405e-06 | -1.62747536503982 |
| LRRC19 | 2.08577233273405e-06 | -1.86310311844674 |
| FXYD3 | 2.17519567460606e-06 | -1.60573400302579 |
| PLCD3 | 2.21177040062813e-06 | -1.4000201948423 |
| SLC22A5 | 2.32596982375221e-06 | -1.35008933734025 |
| PCSK5 | 2.64441638955482e-06 | -1.79497002832944 |
| PBLD | 2.66281307887453e-06 | -1.42793209819708 |
| HIGD1A | 2.72823698821645e-06 | -1.36832111835018 |
| P2RX4 | 2.85472464702383e-06 | -1.06934635927976 |
| GREM2 | 2.87324444511513e-06 | -1.45256245768685 |
| TMPRSS2 | 2.91846804867441e-06 | -1.25408524287385 |
| SLC1A1 | 2.91846804867441e-06 | -1.21549622183352 |
| ATP8A1 | 2.99065687366966e-06 | -1.35954489418376 |
| TMEM61 | 3.31131792831478e-06 | -1.24516655341538 |
| KIAA0513 | 3.36867827340951e-06 | -1.13753692489058 |
| MGLL | 3.36867827340951e-06 | -1.00395002942638 |
| FGFR2 | 3.46060969279208e-06 | -1.41521685901956 |
| FCGRT | 3.58345249556285e-06 | -1.08124221752779 |
| FAM107B | 3.77241982733194e-06 | -1.05184966965627 |
| ACVRL1 | 3.85688885176195e-06 | -1.0254037441955 |
| RDH5 | 4.05368936122912e-06 | -1.18082735214552 |
| LGR4 | 4.08197353768747e-06 | -1.15516863934647 |
| EPB41L4A | 4.10053249665257e-06 | -1.15461801425323 |
| FRMD1 | 4.14135150435427e-06 | -1.29902253406284 |
| NPY1R | 4.18842779337944e-06 | -1.58085780631128 |
| FIGF | 4.72906391209871e-06 | -1.12269833102485 |
| ATP13A4 | 4.77868429878219e-06 | -1.39533289599882 |
| ADH1A | 4.80851156722496e-06 | -2.11450108711508 |
| FUCA1 | 4.8278969691533e-06 | -1.17037215083036 |
| PAPSS2 | 4.85614828742299e-06 | -1.36472939185549 |
| PDE1C | 4.94045385460985e-06 | -1.09840091760399 |
| SEPP1 | 5.12335605501372e-06 | -2.20417407789798 |
| SECTM1 | 5.28853033186777e-06 | -1.38527406105024 |
| ZNF135 | 5.29840469691759e-06 | -1.1645078385197 |
| B3GALT5 | 5.33593678692974e-06 | -1.35211532064969 |
| IL6R | 5.62078333258822e-06 | -1.06266772191021 |
| P2RY14 | 6.16014256672977e-06 | -1.56898920211308 |
| GDPD3 | 6.16014256672977e-06 | -1.59914721212368 |
| NCAM1 | 6.21664552025851e-06 | -1.01802080357336 |
| PAG1 | 6.43083690764405e-06 | -1.0924953898087 |
| PADI2 | 6.45084545933212e-06 | -1.84896972550267 |
| CHRDL1 | 6.68249609120534e-06 | -1.6104283723066 |
| RHOU | 6.97930671119282e-06 | -1.08761324894346 |
| DST | 6.99611374751061e-06 | -1.0433761445957 |
| CASP5 | 7.3090313737465e-06 | -1.23080732235323 |
| AKAP6 | 7.33294671536331e-06 | -1.12568639700836 |
| CYP2C9 | 7.33705654342568e-06 | -1.8539842288173 |
| PRKAR2B | 7.51804544850351e-06 | -1.15748859762489 |
| CMA1 | 7.56852002688114e-06 | -1.06617100501374 |
| MTM1 | 7.57326892692372e-06 | -1.01225931094118 |
| MAMDC2 | 7.58864963869293e-06 | -1.53909000080725 |
| TSPAN1 | 7.73069841794314e-06 | -1.73866059989047 |
| BMP5 | 8.20351428950727e-06 | -1.66715555188404 |
| FXYD1 | 8.49650556785318e-06 | -1.09454820900069 |
| CLMN | 8.50553025575092e-06 | -1.16410570291294 |
| ANGPTL1 | 8.84916221831757e-06 | -1.21796118180895 |
| CLIC5 | 8.95166839939253e-06 | -1.41729845372554 |
| MATN2 | 9.31149989116424e-06 | -1.29783703863869 |
| SLC36A1 | 9.326660928346e-06 | -1.03056595055611 |
| ANK2 | 9.59402667103358e-06 | -1.33055959760095 |
| SELENBP1 | 9.83983387897328e-06 | -1.70756399576435 |
| HSD3B2 | 1.04305564987989e-05 | -2.66806426671263 |
| AKR1B10 | 1.04626026025554e-05 | -2.27339017597057 |
| SLC16A7 | 1.074082380307e-05 | -1.05687554854168 |
| SI | 1.09852137260524e-05 | -2.8072668706531 |
| ISX | 1.11228832076348e-05 | -1.75855478953824 |
| VIP | 1.11228832076348e-05 | -2.17377604957104 |
| ETHE1 | 1.11228832076348e-05 | -1.11814747284052 |
| SCN7A | 1.12546496014094e-05 | -1.80523191051913 |
| SLC6A7 | 1.13809147101377e-05 | -1.07268907728827 |
| DSC2 | 1.13809147101377e-05 | -1.22789375705297 |
| MOGAT2 | 1.14376369032735e-05 | -1.78784516316782 |
| BMX | 1.15383602578449e-05 | -1.1339797863086 |
| ASB2 | 1.16756277860937e-05 | -1.05969071040111 |
| GPR15 | 1.16869145905042e-05 | -1.85041511352023 |
| P2RY1 | 1.17235034827212e-05 | -1.24496903409262 |
| RAVER2 | 1.17367789075308e-05 | -1.11655017733283 |
| SRI | 1.22503626715605e-05 | -1.02073502480504 |
| ZNF334 | 1.24650502805993e-05 | -1.15128264884745 |
| TEX11 | 1.27364783326956e-05 | -1.3270950592022 |
| MAPK10 | 1.28719812556663e-05 | -1.36179238057781 |
| THRB | 1.3032312157875e-05 | -1.21052130313116 |
| FSIP2 | 1.34018039203582e-05 | -1.16807355865004 |
| PLCL2 | 1.36451889912969e-05 | -1.08306405743483 |
| CPNE8 | 1.41384236832393e-05 | -1.37080893240115 |
| CCL28 | 1.42981187445182e-05 | -1.5718724433052 |
| MT1H | 1.51475493347367e-05 | -1.33716648599132 |
| HAPLN1 | 1.51825894666408e-05 | -1.10466851873514 |
| MALL | 1.61388727953788e-05 | -1.44698078078999 |
| SLC9A2 | 1.65164831528274e-05 | -2.02403739393636 |
| CAMK2N1 | 1.78210280108653e-05 | -1.26054213207594 |
| ACADS | 2.00211559262111e-05 | -1.17221528801712 |
| RGS9 | 2.06183606209819e-05 | -1.00618092406421 |
| CAPN13 | 2.06183606209819e-05 | -1.6364366692911 |
| CHGA | 2.1023997535492e-05 | -1.99225050507661 |
| MEP1B | 2.11347272438244e-05 | -1.95188636437492 |
| LIFR | 2.30990850337445e-05 | -1.97328258688146 |
| TTLL7 | 2.34025113182974e-05 | -1.20627059393723 |
| CA12 | 2.46791959576433e-05 | -1.89599676810285 |
| MT1F | 2.50794625155417e-05 | -1.39493179518451 |
| ABCA6 | 2.50794625155417e-05 | -1.23403206798286 |
| NRG4 | 2.56943229731507e-05 | -1.06121758077078 |
| SLC6A19 | 2.58068917770175e-05 | -1.87961114299244 |
| PTPRR | 2.58202018242653e-05 | -1.48740624282306 |
| ADAMDEC1 | 2.58499409139589e-05 | -2.11449420512373 |
| PRKACB | 2.80010198187951e-05 | -1.32864148382538 |
| STAP2 | 2.84953931223334e-05 | -1.06563396925711 |
| MT1A | 2.87075561098338e-05 | -1.11256518448975 |
| MT1M | 2.91131153426677e-05 | -1.42389631302981 |
| TJP3 | 3.01207579106843e-05 | -1.04527553778685 |
| LGALS4 | 3.0929439335397e-05 | -1.83866283438414 |
| GALNT12 | 3.29030475146049e-05 | -1.14167718457907 |
| REP15 | 3.38721078363072e-05 | -2.05124267938467 |
| MAOA | 3.39564585820981e-05 | -1.2452040818714 |
| SLC9A3 | 3.4459488380039e-05 | -2.55958812219952 |
| ATOH1 | 3.50064126452195e-05 | -1.97541719334552 |
| OGN | 3.67695491922954e-05 | -2.27036731922998 |
| CACNB2 | 3.76535408122208e-05 | -1.00903279364855 |
| SHROOM3 | 3.88879810009352e-05 | -1.03836251106881 |
| SLC15A2 | 4.09386908937311e-05 | -1.35134053050159 |
| ESR2 | 4.14792260132118e-05 | -1.20932395907337 |
| CAPN9 | 4.20433495660095e-05 | -1.92983582464969 |
| UNC5C | 4.23579469062962e-05 | -1.05621323252264 |
| XDH | 4.41255133492241e-05 | -1.28438111558858 |
| RBMS3 | 4.60134717997386e-05 | -1.07174758911449 |
| PRIMA1 | 4.86408958312955e-05 | -1.17801616488544 |
| LGALS2 | 5.03985412105459e-05 | -1.21084510612519 |
| TMEM45B | 5.11481495547937e-05 | -1.29590919151956 |
| NOVA1 | 5.38935090695643e-05 | -1.12310288670293 |
| ADH1B | 5.64471393105707e-05 | -2.03974810104446 |
| CNR1 | 5.65140359652811e-05 | -1.12057985258185 |
| ETNK1 | 5.85345339605865e-05 | -1.08797531494328 |
| NEU4 | 5.87977580696908e-05 | -1.3173508600639 |
| MAS1L | 6.02111236860952e-05 | -1.1684611838133 |
| KCNT2 | 6.03431215002159e-05 | -1.13170870044295 |
| SLC41A2 | 6.52682147746607e-05 | -1.01577839431027 |
| GNA11 | 6.63698133754674e-05 | -1.07947476425423 |
| ITM2C | 6.92515855192866e-05 | -1.18850604226343 |
| LRRC31 | 6.95572763511802e-05 | -1.5326545636915 |
| ABI3BP | 6.97146477627131e-05 | -2.11162005530412 |
| IGSF9 | 7.18971205411275e-05 | -1.00937107498602 |
| PDK4 | 7.3619332224144e-05 | -1.80091050179114 |
| EPB41L3 | 7.535339019576e-05 | -1.30906335151598 |
| GCNT3 | 7.74170342452685e-05 | -2.18817410021513 |
| CGN | 7.90102649995193e-05 | -1.27679028173637 |
| SGK2 | 7.91463123360765e-05 | -1.23234961420878 |
| SCNN1A | 8.07031394711986e-05 | -1.25483339490333 |
| CYP2C19 | 8.11130521330114e-05 | -1.22125405358036 |
| ITGA8 | 8.13065333129772e-05 | -1.11281737124166 |
| SLC23A1 | 8.35107871292828e-05 | -1.04521465269124 |
| HMGCS2 | 8.48385246377144e-05 | -2.36383170726485 |
| DEFB1 | 8.97978196088421e-05 | -1.19903789750856 |
| EPHA4 | 9.0926445253709e-05 | -1.01640244471247 |
| SLC16A9 | 9.9462410725738e-05 | -1.45040102602999 |
| PLS1 | 0.000101061029582563 | -1.20586253927651 |
| RPS6KA6 | 0.00010289765916194 | -1.4731752901475 |
| TRPM4 | 0.000108970522894719 | -1.05996141737709 |
| NR1H4 | 0.000111318892134261 | -1.93877284813679 |
| SSBP2 | 0.000111605002790171 | -1.26484218280283 |
| C10orf99 | 0.000113540348608349 | -1.7860242400606 |
| TFCP2L1 | 0.000117506621166678 | -1.24452384629245 |
| LIPH | 0.000118603542459281 | -1.23456816918038 |
| ITPKA | 0.000122335867916673 | -1.1951001818717 |
| NTRK2 | 0.000125553475766836 | -1.01547488711628 |
| EDIL3 | 0.000130960171972654 | -1.03592503093678 |
| FOXP2 | 0.000131975751504507 | -1.37935158432652 |
| GCNT4 | 0.000132319392634199 | -1.03750503833584 |
| B3GALT1 | 0.000132497241793458 | -1.45336303848007 |
| B3GNT6 | 0.000136062251732584 | -1.42807169611565 |
| ITLN1 | 0.000140576193074983 | -2.51115381024155 |
| TOX | 0.000142531268047055 | -1.27006955210885 |
| SLC44A4 | 0.000144663462763266 | -1.41253458168185 |
| IGSF10 | 0.000144949384143956 | -1.20342615943832 |
| SLC20A1 | 0.000145927774901415 | -1.32374613717622 |
| BTNL8 | 0.000152876238399147 | -1.67323282843378 |
| PDZK1 | 0.000162713968282503 | -1.0550196563251 |
| CLU | 0.000165262028188409 | -1.22548799762934 |
| ACVR1C | 0.000168028597201922 | -1.07631707736463 |
| TINAG | 0.000168699946796762 | -1.34084942501923 |
| AQP12A | 0.000170688812711323 | -1.05123652296378 |
| DMRTA1 | 0.000170913503561091 | -1.19526621537992 |
| CMBL | 0.000181663471036712 | -1.38330099422973 |
| C4orf19 | 0.000183322023804098 | -1.15532380688363 |
| LILRB5 | 0.000185500543576855 | -1.08728597123684 |
| ADAM28 | 0.000188323936814634 | -1.17402409822336 |
| VAV3 | 0.000191815554283804 | -1.04379134809941 |
| TST | 0.000192823045012106 | -1.32719078482249 |
| SOX6 | 0.000197714292434151 | -1.13670090764655 |
| XKR4 | 0.000205182346938235 | -1.18920369023579 |
| C1orf106 | 0.000210359305561564 | -1.0068217750033 |
| IL1R2 | 0.000214713811549848 | -1.01234002098236 |
| CLEC3B | 0.000221704528367617 | -1.04619184976597 |
| SCGB2A1 | 0.000226193438255765 | -1.49976240732625 |
| CREB3L3 | 0.000228418576824972 | -1.05034340441414 |
| C19orf33 | 0.00022945831738731 | -1.20792543685063 |
| MYO1A | 0.000233005269683567 | -1.2047308235183 |
| MAB21L1 | 0.000233083795974009 | -1.03314044463793 |
| SPIB | 0.000238553794796679 | -1.66181667323084 |
| UGDH | 0.000241095030728753 | -1.05484873731927 |
| MT1X | 0.000248001421377085 | -1.05162854873946 |
| SFRP1 | 0.000252218531973874 | -1.2192986556217 |
| PPP1R14D | 0.00025534112731878 | -1.00315843209869 |
| HHIP | 0.0002670739197782 | -1.23318486546929 |
| FAM3D | 0.000267428438818754 | -1.37295073702305 |
| RETNLB | 0.000270809228725096 | -1.76007339911394 |
| GPA33 | 0.000275257424460564 | -1.46956113013036 |
| MUC20 | 0.000287499751329657 | -1.12929340249626 |
| NRG1 | 0.000289390151336365 | -1.1596719423687 |
| KLHDC1 | 0.000306960120307053 | -1.11927576293578 |
| RAPGEFL1 | 0.000310854085149271 | -1.06824800623604 |
| RNF125 | 0.000321644315447708 | -1.04780028072593 |
| AKAP5 | 0.000328167647395688 | -1.02328747911017 |
| KCNA3 | 0.000345696619442119 | -1.52590192261808 |
| PTGDR | 0.000356871160789386 | -1.3468901351383 |
| MT1G | 0.000357467884192414 | -1.68500153583747 |
| FABP2 | 0.000359185391043884 | -2.10568229807656 |
| FABP1 | 0.000381524062954717 | -2.2218066047418 |
| FLT3 | 0.000383330699201026 | -1.09151996298001 |
| LRMP | 0.000398848013797102 | -1.32614918860551 |
| AFF3 | 0.000420570563403451 | -1.19503945857374 |
| WDR78 | 0.00044911140311893 | -1.04136329871287 |
| GHR | 0.000553463246221461 | -1.12833463577445 |
| BCHE | 0.000568536359718529 | -1.2899475729965 |
| CD163L1 | 0.000578914256575537 | -1.17491503148344 |
| JAM2 | 0.000595688655478054 | -1.31307585828696 |
| HSD3B1 | 0.000595688655478054 | -1.16763669710569 |
| CKMT1A | 0.000816590425579707 | -1.19078897813132 |
| FCER1A | 0.000827707808658636 | -1.01354813748535 |
| CYP2C18 | 0.000872666069745536 | -1.34170295640033 |
| ABCC3 | 0.000877057318286703 | -1.08914797383951 |
| NR3C1 | 0.000904452059342911 | -1.10522569122941 |
| HRASLS2 | 0.000905971622052255 | -1.05874801884931 |
| SYNPO2 | 0.000922173161335447 | -1.09136158977985 |
| FHL5 | 0.00095358763998959 | -1.00449586992427 |
| TMEM54 | 0.000998245665968481 | -1.09908074377213 |
| MFAP4 | 0.000999050675521308 | -1.11026812107524 |
| FRZB | 0.00103716690705859 | -1.09819508522672 |
| MUC4 | 0.00104579118845887 | -1.37602236013804 |
| BTLA | 0.00117531709569023 | -1.17907272321067 |
| ATP1A2 | 0.00126138461377287 | -1.31926565636599 |
| KRT19 | 0.00143233050780035 | -1.21335107583784 |
| CYP3A5 | 0.00149170420451115 | -1.12369326257643 |
| SLITRK3 | 0.00152162972053506 | -1.04931541326183 |
| DQX1 | 0.0016305272459928 | -1.20920820374675 |
| F13A1 | 0.0018655876065478 | -1.13650301484079 |
| FGL2 | 0.0018817085200282 | -1.21002378124084 |
| ABCB1 | 0.00188931702656392 | -1.18610052133178 |
| APOBEC3B | 0.00198942726763782 | -1.03616105026879 |
| SRPX | 0.00202822621236902 | -1.1061425673484 |
| MCOLN2 | 0.00204380891269191 | -1.09095425843295 |
| SATB2 | 0.00223178831413166 | -1.05468432388432 |
| C2orf40 | 0.00234794741707974 | -1.08035090696075 |
| GNG7 | 0.00235113638659923 | -1.00960379078884 |
| SERINC2 | 0.00240255186546037 | -1.08739327094405 |
| MAL | 0.00263023778309833 | -1.03178219185954 |
| PROM1 | 0.00266891151860951 | -1.39287604435203 |
| GCG | 0.00270364922180165 | -1.67730173683971 |
| TNFRSF17 | 0.00282406300246443 | -1.58173630219806 |
| CYP2C8 | 0.0029248448685414 | -1.10200910362088 |
| UGT2B11 | 0.00299481967906751 | -1.037703705422 |
| CD180 | 0.00327833644711581 | -1.14634730504315 |
| MMRN1 | 0.00329224431382065 | -1.12083334584444 |
| C7 | 0.00334484394956791 | -1.38922752304504 |
| SLC9A9 | 0.00340444549951679 | -1.12736212593451 |
| CNTFR | 0.00369573185121053 | -1.05152416851085 |
| PI16 | 0.00396109062515314 | -1.15259100432897 |
| CR2 | 0.00420015836334275 | -1.64871766913832 |
| KNG1 | 0.00435406864224568 | -1.03665766019328 |
| CEACAM1 | 0.00444051825418226 | -1.20007973131342 |
| COLEC12 | 0.00453249901703437 | -1.07098333345632 |
| PCDH20 | 0.00453394930184651 | -1.01075055254047 |
| LMO3 | 0.00458388604171487 | -1.03707103152959 |
| EDN2 | 0.00475633724221848 | -1.04400778659627 |
| DNASE1L3 | 0.00546258934402526 | -1.21459447555927 |
| MFAP5 | 0.00564299014831667 | -1.08042685484114 |
| ADIPOQ | 0.0060148462087764 | -1.13863677754257 |
| PIGR | 0.00605688861954908 | -1.73643007775707 |
| KRTAP13-2 | 0.00629708953601073 | -1.00845929810433 |
| GALNT8 | 0.0065134006325349 | -1.06711886199381 |
| COL17A1 | 0.00671330241657905 | -1.20811828647665 |
| HTR4 | 0.00830867292178559 | -1.03400847620979 |
| KLRB1 | 0.0117266512300265 | -1.23185129402305 |
| LEFTY1 | 0.0120183114898453 | -1.15065359521514 |
| LTF | 0.0121727130943919 | -1.06126512276025 |
| CALB2 | 0.0122698229615138 | -1.25373434355164 |
| FABP4 | 0.0129860652596354 | -1.43730333680932 |
| TRIM31 | 0.0157021581289906 | -1.17173519753488 |
| AKR1C3 | 0.0166187893205226 | -1.13153142399099 |
| SLITRK6 | 0.022934539258777 | -1.40355584499393 |
| CD96 | 0.0248003883522201 | -1.03932331768219 |
| LAX1 | 0.0324837655259132 | -1.05897957298341 |
| AGR3 | 0.0429397030001457 | -1.04839806199975 |
| SLC38A4 | 0.0445469080269846 | -1.07595151149768 |
| DES | 0.0485332972479 | -1.04393870502401 |

**Upregulated DEmRNAs**

| Symbol | Adjusted P.value | Log2FC |
| --- | --- | --- |
| IFITM1 | 1.70137291582144e-12 | 1.84952958822008 |
| IFITM2 | 2.15298143661504e-12 | 1.72950943030985 |
| IFITM3 | 1.30249276145429e-11 | 1.99031389413179 |
| TGFBI | 3.54833437548077e-10 | 2.33207236694355 |
| SLC7A5 | 3.54833437548077e-10 | 2.00662711884648 |
| CALU | 3.76734620716036e-10 | 1.24807996314318 |
| MTHFD1L | 4.26219520224409e-10 | 1.34910568125868 |
| GRAMD1A | 4.65635365090732e-10 | 1.19325313526249 |
| CLDN1 | 5.25282426604664e-10 | 3.16014647587768 |
| HSP90AB1 | 5.43772600476653e-10 | 1.01997886095496 |
| TIMP1 | 1.03905330133933e-09 | 1.96624032478188 |
| CTHRC1 | 1.07184057866666e-09 | 2.40445626303609 |
| CDH3 | 1.95717224518614e-09 | 2.21342247611724 |
| CDK4 | 2.64133741843165e-09 | 1.3054554904075 |
| COL4A1 | 3.87720746694476e-09 | 1.55123829675832 |
| PPAT | 6.15434014359221e-09 | 1.07904986531141 |
| DKC1 | 6.28521161022659e-09 | 1.18246273163912 |
| CDC25B | 6.69438622554269e-09 | 1.37126880151697 |
| DCUN1D5 | 8.69154272550336e-09 | 1.21912119601292 |
| MYC | 1.01974186185289e-08 | 1.52029262370212 |
| SRM | 1.06676347638231e-08 | 1.06390374669944 |
| RIPK2 | 1.21512026030744e-08 | 1.06718007022405 |
| GTF3A | 1.32563012934752e-08 | 1.23459977224829 |
| SNRPF | 1.32563012934752e-08 | 1.17565701801119 |
| GRINA | 1.56340777861956e-08 | 1.10153933707144 |
| STC2 | 1.60461991525535e-08 | 2.28183864483494 |
| PA2G4 | 1.71415696045786e-08 | 1.01776138025558 |
| S100A11 | 1.9929335423914e-08 | 1.66031924219112 |
| RAN | 2.06400261739563e-08 | 1.04152491911514 |
| CKAP2 | 2.71421990243086e-08 | 1.25669951814809 |
| PPIL1 | 2.84825816495719e-08 | 1.11779079221511 |
| VSNL1 | 2.96682547945376e-08 | 1.83457428207154 |
| THY1 | 3.18216950477237e-08 | 1.53212481140554 |
| PDCD2L | 3.18216950477237e-08 | 1.27786810844963 |
| SCD | 3.25706734438995e-08 | 1.93692099938186 |
| RGS16 | 3.40521171546691e-08 | 1.46522904987976 |
| BGN | 4.14005156616797e-08 | 2.26454611606707 |
| COL12A1 | 4.14005156616797e-08 | 1.84674527432596 |
| COL1A2 | 4.67746874917829e-08 | 1.45616581479961 |
| SLC29A1 | 4.67746874917829e-08 | 1.57268767287284 |
| CSE1L | 5.67424709401516e-08 | 1.28784425326583 |
| TRIB3 | 5.76144678001156e-08 | 1.67136463587194 |
| CCT2 | 5.807550102698e-08 | 1.02968125718459 |
| LYAR | 5.96361214049169e-08 | 1.16346205991727 |
| NFE2L3 | 7.76507133811206e-08 | 1.53683813080552 |
| ZAK | 7.81597890206707e-08 | 1.33915087764079 |
| RUVBL1 | 7.81597890206707e-08 | 1.3533732248934 |
| RNASEH2A | 8.16726001502053e-08 | 1.01854001124537 |
| NOLC1 | 8.31758187425617e-08 | 1.00735992084124 |
| PLAU | 9.55967592447311e-08 | 1.67291516060179 |
| MMP11 | 9.95114608858672e-08 | 1.59912280617237 |
| LY6E | 1.0764133532718e-07 | 1.81848500863316 |
| LRP8 | 1.1107866937793e-07 | 1.86375482656085 |
| PHLDA1 | 1.12614842415551e-07 | 1.06704419062181 |
| NME1 | 1.23129397376975e-07 | 1.09431774445231 |
| WISP1 | 1.23129397376975e-07 | 1.72290096424821 |
| DDX21 | 1.42540891186915e-07 | 1.27646383733251 |
| PEA15 | 1.46447814785144e-07 | 1.07458997617846 |
| TOMM34 | 1.47787017120586e-07 | 1.48071676267293 |
| GRIN2D | 1.49117390895834e-07 | 1.86673540672891 |
| CDK2 | 1.55341177559119e-07 | 1.04662205071142 |
| DUSP14 | 1.63934090360826e-07 | 1.21222927311987 |
| ATP6V1E2 | 1.79594036737899e-07 | 1.12475926428772 |
| SNAI1 | 1.79735324037656e-07 | 1.1990915731786 |
| ABCE1 | 1.82581155568081e-07 | 1.1505199525155 |
| PRDX4 | 1.88389839738397e-07 | 1.29453078627514 |
| CHCHD6 | 1.88389839738397e-07 | 1.25375815005646 |
| AGTRAP | 1.94868775662989e-07 | 1.09834078647663 |
| MMP7 | 2.19314355683138e-07 | 3.15565877217777 |
| TRAP1 | 2.19314355683138e-07 | 1.07981766563561 |
| TRIP13 | 2.21865600835039e-07 | 1.57813996771597 |
| PSAT1 | 2.21865600835039e-07 | 1.48163428195356 |
| DGAT2 | 2.43530447162156e-07 | 1.14627606942786 |
| COL8A1 | 2.5562580422977e-07 | 1.60326579984125 |
| GPR180 | 2.87567557719211e-07 | 1.19426750563437 |
| SLC39A10 | 2.87567557719211e-07 | 1.17486250595231 |
| PPT1 | 3.13008968515745e-07 | 1.0145291872822 |
| MID1IP1 | 3.54505836026125e-07 | 1.03346875791681 |
| CST1 | 3.59334267864063e-07 | 3.59730569982829 |
| CKS2 | 3.80550308705175e-07 | 1.57096518096806 |
| SUV39H1 | 3.83506619326866e-07 | 1.08745141148354 |
| EEF1E1 | 4.16572065720026e-07 | 1.0173306179606 |
| FADS2 | 4.17724109352525e-07 | 1.20794444893284 |
| POLD2 | 4.51770898206217e-07 | 1.17654942606137 |
| PALM2 | 4.64797295533265e-07 | 1.27775564185988 |
| PUS7 | 4.90537168941004e-07 | 1.2719069609644 |
| HSPE1 | 4.94908351003781e-07 | 1.09817901241447 |
| SULF1 | 5.14059122817668e-07 | 1.9957719645129 |
| TMPRSS3 | 5.24719552907807e-07 | 1.74112890122766 |
| THBS2 | 5.63855862581458e-07 | 2.22102527294365 |
| MIF | 5.68862535431224e-07 | 1.08890267052702 |
| EDNRA | 6.0268026562251e-07 | 1.35788892763744 |
| PDPN | 6.10967135582722e-07 | 1.46560010777873 |
| WDR4 | 6.3240597866228e-07 | 1.06238497261289 |
| NUDCD1 | 6.3240597866228e-07 | 1.06097504536801 |
| CHAF1B | 6.83198812004062e-07 | 1.23457176357641 |
| SHMT2 | 7.71412809673428e-07 | 1.05596377943579 |
| ADA | 8.13955383340949e-07 | 1.06820828907072 |
| ESM1 | 8.3495588588056e-07 | 1.10595129859736 |
| ECT2 | 8.48701154476437e-07 | 1.43249585839887 |
| MSX1 | 8.48701154476437e-07 | 1.17781800716719 |
| HOMER1 | 8.64443059027028e-07 | 1.03462854096898 |
| KLK6 | 9.43787724026772e-07 | 1.65261143000593 |
| DUSP4 | 9.53617944622893e-07 | 1.30113928015267 |
| HSPD1 | 9.59369852522322e-07 | 1.14052770567546 |
| COL5A2 | 1.0731632414453e-06 | 1.16303689542496 |
| FXYD5 | 1.08068461138263e-06 | 1.15145341834575 |
| PTP4A3 | 1.11783845237085e-06 | 1.09926928618859 |
| ADAMTS12 | 1.23842618383571e-06 | 1.82483033627196 |
| PAICS | 1.25727557469921e-06 | 1.10347973757386 |
| COL6A3 | 1.27000558402476e-06 | 1.47792544426554 |
| KIAA1199 | 1.27176770607741e-06 | 2.15117770553453 |
| EXO1 | 1.35943354808142e-06 | 1.04938566592043 |
| SSB | 1.41825940218008e-06 | 1.06478709038203 |
| C9orf116 | 1.57352115941408e-06 | 1.02711044785389 |
| GINS1 | 1.5832617462712e-06 | 1.39295310361334 |
| TGIF2 | 1.60787502481173e-06 | 1.13144536786817 |
| ENC1 | 1.61560722810647e-06 | 1.4373782779092 |
| STIP1 | 1.63599630819358e-06 | 1.00566678911803 |
| CXCL11 | 1.81412617419494e-06 | 2.68722976969457 |
| ANLN | 1.81825249477375e-06 | 1.22508049433205 |
| RAD51AP1 | 1.98981449988304e-06 | 1.15359833557752 |
| CCND1 | 2.05373100512092e-06 | 1.26140368180173 |
| FAP | 2.1746407216848e-06 | 2.5659359236426 |
| GRIN2B | 2.2422643403139e-06 | 1.54552355516341 |
| MMP1 | 2.33650783743525e-06 | 2.33029345861526 |
| KRT80 | 2.6153862168934e-06 | 1.7753202048803 |
| PLK1 | 3.12731097813202e-06 | 1.1523650075938 |
| S100A9 | 3.53291352581618e-06 | 1.65268284447639 |
| CST2 | 3.55596695929266e-06 | 2.55181098793995 |
| LDHB | 3.76755184699079e-06 | 1.06970546133457 |
| SNTB1 | 3.81520111050308e-06 | 1.1601856932818 |
| AQP9 | 3.87097303678423e-06 | 1.71190893657433 |
| PCNA | 4.28682966279308e-06 | 1.04566609521044 |
| DTL | 4.28936024991649e-06 | 1.13447291546377 |
| EGFL6 | 4.33480192219176e-06 | 1.60163287435957 |
| PPA1 | 4.66223810863799e-06 | 1.01081796641643 |
| COL1A1 | 4.70090482924527e-06 | 1.66938154072595 |
| IARS | 4.77868429878219e-06 | 1.02953970303596 |
| TOP2A | 4.85614828742299e-06 | 1.21474367601007 |
| FANCB | 5.12807385534007e-06 | 1.00868380050782 |
| STAT1 | 5.42019754230837e-06 | 1.13377125757133 |
| MMP3 | 5.58503331923853e-06 | 2.42631968922202 |
| CDCA7 | 5.778001567417e-06 | 1.07726710173082 |
| PDGFRB | 5.89507461557014e-06 | 1.20152230149758 |
| TPX2 | 6.1881973482074e-06 | 1.33959984307863 |
| SLC7A11 | 6.34978969788313e-06 | 1.24496150053171 |
| GARS | 6.36251434683092e-06 | 1.00502101728979 |
| RRS1 | 6.76064576229996e-06 | 1.09073650475714 |
| NKD1 | 7.12671639629406e-06 | 1.66030312579614 |
| MFAP2 | 7.12671639629406e-06 | 1.29134683348478 |
| ARNTL2 | 7.1849444186106e-06 | 1.46384822908778 |
| UBE2T | 7.33696347049596e-06 | 1.27378002442407 |
| SLC6A6 | 7.58044664822363e-06 | 1.18652316618738 |
| FOXQ1 | 7.60555507568863e-06 | 1.89304349596255 |
| GTF2IRD1 | 7.73069841794314e-06 | 1.17196158570207 |
| COL5A1 | 7.84981690673868e-06 | 1.11463458893338 |
| UBE2C | 7.91698122717497e-06 | 1.30534382870569 |
| CXCL10 | 8.174217827595e-06 | 1.74486710978678 |
| IMMP2L | 8.62027799942783e-06 | 1.01070999157258 |
| HAPLN3 | 9.45956453915018e-06 | 1.01922524243059 |
| MAD2L1 | 9.71528248757742e-06 | 1.44430926781397 |
| FKBP10 | 1.00067314574049e-05 | 1.11361530781324 |
| INHBA | 1.06025683792724e-05 | 1.40805124997749 |
| CHEK1 | 1.06356732098651e-05 | 1.10786497701619 |
| MMP12 | 1.09250014705786e-05 | 1.98347102067735 |
| NOX4 | 1.09364854115591e-05 | 1.20352320543179 |
| CFB | 1.10535640779749e-05 | 1.45339808159664 |
| CCNF | 1.14234956292496e-05 | 1.21101016006863 |
| GRHL1 | 1.14372172217456e-05 | 1.12079467162261 |
| NUF2 | 1.24671440135147e-05 | 1.17477304315024 |
| COL10A1 | 1.28611919783907e-05 | 1.59352018882831 |
| PRSS22 | 1.28744794602578e-05 | 1.37227762770478 |
| TMEM97 | 1.30946358843844e-05 | 1.46374824026355 |
| KIF2C | 1.41296764570983e-05 | 1.02790838020237 |
| LGR6 | 1.42644933752499e-05 | 1.09348143610106 |
| HSPH1 | 1.45642973396652e-05 | 1.30947693970822 |
| HECW2 | 1.60917681779246e-05 | 1.03546963888947 |
| VCAN | 1.6741281911507e-05 | 1.39245785502987 |
| TNFAIP6 | 1.72927688704506e-05 | 1.72795366695427 |
| PMAIP1 | 1.74462092628764e-05 | 1.0608670003294 |
| KIF14 | 1.84051426161416e-05 | 1.17668996095567 |
| S100A8 | 1.84522433256679e-05 | 2.10103329882329 |
| PRRX1 | 1.8576330615955e-05 | 1.605186263038 |
| ETV4 | 1.91349548425059e-05 | 1.56329575021025 |
| UBD | 1.94482456704484e-05 | 2.13258230652183 |
| CLDN2 | 1.94595547689568e-05 | 1.91044071111399 |
| ZP3 | 2.06183606209819e-05 | 1.05876372327773 |
| GALNT6 | 2.07325175966154e-05 | 1.10906410460642 |
| NT5DC2 | 2.20739468721027e-05 | 1.04244064168451 |
| OLR1 | 2.27133886150046e-05 | 1.69306300042549 |
| OLFML2B | 2.30161539149823e-05 | 1.29631901435033 |
| FJX1 | 2.33444565625474e-05 | 1.1790627749586 |
| SPP1 | 2.34022371696805e-05 | 2.72770000434254 |
| CCNA2 | 2.37305206125642e-05 | 1.02250405515637 |
| FOXM1 | 2.61752150582634e-05 | 1.00714444014833 |
| MPP6 | 2.67804295234766e-05 | 1.09027019070511 |
| ITGA2 | 2.73227556634532e-05 | 1.25696622944238 |
| TNC | 2.85380701114114e-05 | 1.52160384716754 |
| ALDH4A1 | 2.90287576491379e-05 | 1.04943358058407 |
| EIF5A2 | 3.03198252440472e-05 | 1.04173383165952 |
| TNS4 | 3.03992384180726e-05 | 1.78083927048834 |
| STC1 | 3.07190303729763e-05 | 1.21361960939752 |
| MMP9 | 3.15786025018702e-05 | 1.47749696707646 |
| AREG | 3.31275375228929e-05 | 1.1566817071785 |
| EREG | 3.70809627775324e-05 | 1.38987956742841 |
| BOP1 | 3.71449856898002e-05 | 1.04122674368573 |
| IL1RN | 3.7706871048802e-05 | 1.2953667672831 |
| TRIM29 | 3.88225078746028e-05 | 1.96579725915209 |
| AGT | 4.15213329833039e-05 | 1.1085617241208 |
| CHI3L1 | 4.20173185151037e-05 | 1.9648248887444 |
| TSPAN5 | 4.32381600721304e-05 | 1.17421030748887 |
| IGFBP7 | 4.56871549384464e-05 | 1.2030448393393 |
| LGR5 | 5.15914631542468e-05 | 1.50564417988593 |
| TTK | 5.19751742371042e-05 | 1.18721005485173 |
| KIF20A | 5.22742251824341e-05 | 1.12701879410285 |
| COL3A1 | 5.44656872108901e-05 | 1.2519857444826 |
| CEP55 | 5.44656872108901e-05 | 1.2577797101102 |
| LAMP3 | 5.85204006953154e-05 | 1.18591601181453 |
| CDKN3 | 5.99730047118189e-05 | 1.1320231576015 |
| TNFSF15 | 6.01948120066319e-05 | 1.07728877062113 |
| SERPINE2 | 6.07648007675579e-05 | 1.08520683141364 |
| SFRP4 | 6.14955231673618e-05 | 1.95595593160745 |
| SPOCK1 | 6.37446126921961e-05 | 1.04478855624417 |
| FNDC1 | 6.39938446688025e-05 | 1.49908200800734 |
| PYCR1 | 6.62448860141245e-05 | 1.01114189135964 |
| LEF1 | 6.7926528767051e-05 | 1.18205063189509 |
| ADAMTS4 | 6.92179027224744e-05 | 1.15713854629892 |
| CCDC113 | 7.59817476610885e-05 | 1.05834732512211 |
| HUNK | 7.93951070560495e-05 | 1.02098073950928 |
| ASCL2 | 8.38292661980252e-05 | 1.31614326794287 |
| MND1 | 8.41882252589178e-05 | 1.01556714314369 |
| BUB1 | 8.97597410267225e-05 | 1.00775768601229 |
| SLCO4A1 | 9.17198171839725e-05 | 1.42868400563452 |
| ACAN | 9.18754052767421e-05 | 1.05972854527063 |
| CXCL1 | 9.20371999773469e-05 | 1.29238131230786 |
| TNFRSF12A | 9.45498355355101e-05 | 1.25335670796789 |
| SOX14 | 9.94590366130935e-05 | 1.2965061773156 |
| CGREF1 | 0.000100828495486349 | 1.00270464461122 |
| LAPTM4B | 0.000106324475668205 | 1.12592157754017 |
| GOLT1A | 0.000106726893082141 | 1.39602272422843 |
| FN1 | 0.000110577369065119 | 1.36171716355153 |
| MYOM3 | 0.00011200053004716 | 1.22985360027131 |
| TESC | 0.000117407477272657 | 1.63269661242687 |
| SERPINB5 | 0.000117755289522904 | 2.09847360097779 |
| S100A2 | 0.000118603542459281 | 1.2889431249362 |
| VWA2 | 0.00012054307326815 | 1.21776805337797 |
| SCML1 | 0.00012157576007949 | 1.09643553084496 |
| MSX2 | 0.000124525425997724 | 1.1638525740376 |
| WNT2 | 0.000130896439640668 | 1.03097628183144 |
| FOSL1 | 0.00013879456589689 | 1.27668050123206 |
| ITGBL1 | 0.000140863932823931 | 1.00512809556245 |
| CA9 | 0.000141088723969483 | 2.32948945468764 |
| CCNB1 | 0.000161058905732493 | 1.11730156735601 |
| TRIM59 | 0.000177301553213897 | 1.01454937467601 |
| SPARC | 0.000177706255897191 | 1.10588346744629 |
| CENPF | 0.000182670741861823 | 1.01098668621765 |
| IFI6 | 0.000186635703181998 | 1.29122386349456 |
| RNF43 | 0.000189423137244953 | 1.27120863240437 |
| SLC12A2 | 0.000192912302764984 | 1.17466835147862 |
| SERPINE1 | 0.000209486653615554 | 1.55081434368941 |
| FCGR3A | 0.000210359305561564 | 1.2172714073973 |
| MET | 0.000226676090807107 | 1.2434113522131 |
| MYBL2 | 0.000231371420493146 | 1.06609243076413 |
| CXCL9 | 0.000260845901358278 | 1.38017880380768 |
| BMP7 | 0.000265159113766438 | 1.13670733030589 |
| HTR1D | 0.000278711041712967 | 1.17202087643348 |
| MXRA5 | 0.000290189002455626 | 1.23999660298367 |
| KIF18A | 0.000290378801793361 | 1.08008354427569 |
| IL1A | 0.000341565572857522 | 1.12402430006237 |
| SELE | 0.00035131402027097 | 1.17501323154323 |
| DPEP1 | 0.000354611320624754 | 1.4193737710659 |
| SQLE | 0.000356438658019432 | 1.08998640297291 |
| F2RL2 | 0.000359185391043884 | 1.08976884510797 |
| SP5 | 0.000377664230976957 | 1.02955490082041 |
| TACSTD2 | 0.000378373972581844 | 1.54591419945477 |
| AZGP1 | 0.000389617922655804 | 1.09808758138351 |
| LRRC15 | 0.000418281072646 | 1.28322217562319 |
| CXCL2 | 0.000423124402080802 | 1.03076613166105 |
| RPL39L | 0.000464949131965973 | 1.2925229929732 |
| KCNJ15 | 0.000580690551686336 | 1.10424142864515 |
| NT5DC3 | 0.000582851287011793 | 1.08916504175301 |
| DHCR7 | 0.000806803134722459 | 1.03672070343901 |
| SRPX2 | 0.000808440778884776 | 1.20744452984506 |
| FSCN1 | 0.000844624555344108 | 1.05282683143978 |
| SLC35D3 | 0.000902137454381258 | 1.26780048036146 |
| IRX3 | 0.000998245665968481 | 1.48580456502625 |
| ZIC2 | 0.000998247723298975 | 1.53916871779502 |
| PCSK9 | 0.00121608176235625 | 1.07303814489485 |
| SLC6A20 | 0.0012856232003196 | 1.31871672630132 |
| BST2 | 0.00137832547319371 | 1.45378550628542 |
| PDX1 | 0.00149170420451115 | 1.1616542734342 |
| NMU | 0.00151381454944943 | 1.17931449280538 |
| OTX1 | 0.00180414839145099 | 1.08828259489307 |
| DKK4 | 0.00226090072478985 | 1.3390192104554 |
| KLK7 | 0.0024875860139134 | 1.6167985950727 |
| OAS2 | 0.00280468582882027 | 1.06095534539198 |
| LUM | 0.00282406300246443 | 1.03312853779554 |
| SULT2B1 | 0.00288822353183006 | 1.02897421189729 |
| HS6ST2 | 0.00316434922761153 | 1.07006616988793 |
| GABRP | 0.00351266082585576 | 1.08373833652332 |
| APOC1 | 0.00356669740579074 | 1.21809195135443 |
| SAA2 | 0.00382402392793539 | 1.2822393298797 |
| IL6 | 0.00402465610008264 | 1.22938699001649 |
| SLC2A3 | 0.00431060623037126 | 1.03979254361139 |
| GJB5 | 0.0045436400269123 | 1.30920781141032 |
| IFI44 | 0.00486661316583534 | 1.11994590726764 |
| CKMT2 | 0.00504766049804583 | 1.29009920516657 |
| TCN1 | 0.00515736235967384 | 1.24294755304738 |
| LCN2 | 0.00527035100981922 | 1.50281336998203 |
| EPHB3 | 0.00565808026845476 | 1.03500062468353 |
| KRT23 | 0.00565808026845476 | 1.55561731005456 |
| LEMD1 | 0.00656360943355435 | 1.37246363439252 |
| ZIC5 | 0.00726826682627115 | 1.11492523983258 |
| ACSL6 | 0.00836092907365763 | 1.05469200269337 |
| IL33 | 0.00871139655167755 | 1.15655447436962 |
| GJB3 | 0.00885087223464784 | 1.03248168088675 |
| ODAM | 0.0089104430727002 | 1.0775708883127 |
| QPCT | 0.00966867572673688 | 1.00847914990651 |
| NMUR2 | 0.0101107706395875 | 1.24644106616648 |
| SLC6A14 | 0.0108957016915017 | 1.05325637322369 |
| S100P | 0.0115868073734337 | 1.52824145285857 |
| SAA1 | 0.0125073517217413 | 1.27584219960387 |
| DUOXA2 | 0.0131761457358731 | 1.07582229723841 |
| CYP4X1 | 0.0182199200940885 | 1.32842212710826 |
| SLCO1B3 | 0.0346881031220777 | 1.31009467813709 |

Table S2. Gene Significances (GS) and module memberships (MM).

| GS | Green MM | Tan MM | Turquoise MM |
| --- | --- | --- | --- |
| AARS | AARS | ABCE1 | AARS |
| ABCE1 | ACSL4 | ACBD6 | ABCE1 |
| ABI2 | ACTN1 | ADAR | ABI2 |
| ACBD6 | ADAM12 | ADNP | ACACA |
| ACLY | ADAM19 | AKAP11 | ACBD6 |
| ACTN1 | ADAMTS12 | AMZ2 | ACD |
| ADA | ADAMTS2 | ANAPC1 | ACLY |
| ADAMTS12 | ADAMTS4 | ANP32B | ACOT9 |
| ADAR | ADAMTS6 | APEX1 | ACTL6A |
| ADCY3 | ADAMTS9 | API5 | ACTN1 |
| AGTRAP | ADAR | ASF1A | ACTR5 |
| AHR | AEBP1 | ASNSD1 | ADAMTS12 |
| ANAPC1 | AGTRAP | ATIC | ADAR |
| ANAPC13 | ANGPT2 | ATP11C | ADCY3 |
| ANLN | ANGPTL2 | BBS10 | ADRM1 |
| AP1S3 | ANTXR1 | BCCIP | AGT |
| APEX1 | ANXA5 | BCOR | AGTRAP |
| AQP9 | APOC1 | BID | AHCY |
| ARMC10 | AQP9 | BTF3 | AHSA1 |
| ARNTL2 | ARMCX6 | BUB3 | ALDH1B1 |
| ASNSD1 | ASPN | C11orf73 | ALDH4A1 |
| ASPHD1 | ATP6V1E2 | C2orf44 | ALG1 |
| ATIC | BCAT1 | C4orf27 | ALG3 |
| ATP6V1E2 | BGN | C5orf28 | ALG5 |
| ATP6V1F | C1R | C6orf48 | ALG8 |
| B3GALT6 | C5AR1 | CAMSAP1 | AMPD2 |
| B3GNTL1 | CALD1 | CCDC59 | ANAPC1 |
| BAG3 | CALU | CCT2 | ANAPC11 |
| BAZ1B | CBFB | CCT4 | ANAPC13 |
| BCL2L12 | CCL22 | CCT6A | ANLN |
| BGN | CCR1 | CCT8 | AP1S1 |
| BUB3 | CD248 | CD47 | AP1S3 |
| C11orf73 | CD81 | CKAP2 | AP2B1 |
| C12orf45 | CD93 | CLNS1A | ARL5B |
| C5orf28 | CDH11 | CPSF3 | ARL8A |
| C9orf116 | CHI3L1 | CPSF6 | ARMC1 |
| CAD | CHN1 | CREG1 | ARMC10 |
| CALU | CHSY1 | CSE1L | ARMC6 |
| CANX | CLEC11A | CSNK2A2 | ARMCX6 |
| CBFB | CMTM3 | CUL1 | ARNTL2 |
| CBX3 | CNN2 | DARS | ARPC5L |
| CCDC74B | COL12A1 | DCUN1D5 | ASF1B |
| CCT2 | COL15A1 | DDX21 | ASNSD1 |
| CCT3 | COL18A1 | DEGS1 | ASPHD1 |
| CCT4 | COL1A2 | DHX9 | ASPM |
| CCT7 | COL3A1 | DKC1 | ATIC |
| CD47 | COL4A1 | DNAJC10 | ATP11A |
| CDC25B | COL5A1 | DPM1 | ATP6V1E2 |
| CDCA7 | COL5A2 | DUSP11 | ATP6V1F |
| CDH11 | COL6A1 | DUSP12 | ATR |
| CDH3 | COL6A3 | EEF1A1 | AURKA |
| CDK2 | COL8A1 | EEF1B2 | AURKB |
| CDK4 | COPZ2 | EEF1D | AZGP1 |
| CEBPZ | CPXM1 | EEF1E1 | B4GALT7 |
| CEP72 | CTHRC1 | EIF4A3 | BACE2 |
| CHAF1B | CTSH | ERH | BAG3 |
| CHCHD6 | CTSK | EXOSC8 | BANF1 |
| CKAP2 | CXCL9 | FAM60A | BAZ1B |
| CKLF | DBN1 | FBXO5 | BCAP31 |
| CKS1B | DCHS1 | FTSJ1 | BCL2L12 |
| CKS2 | DCTD | FXR1 | BFAR |
| CLDN1 | DEGS1 | FXYD5 | BGN |
| CLNS1A | DFNA5 | GARS | BID |
| CNN2 | DKK3 | GLO1 | BMP4 |
| COL12A1 | DNMT1 | GMPS | BMP7 |
| COL1A2 | DPYSL3 | GRAMD1A | BOLA2B |
| COL4A1 | DUSP14 | GRPEL2 | BOP1 |
| COL5A1 | DYSF | GSTO1 | BRCA1 |
| COL5A2 | EDNRA | GTF2H3 | BRCA2 |
| COL6A3 | EFEMP2 | GTF2H5 | BRIP1 |
| COL8A1 | EGFL6 | GTF3A | BUB1 |
| COMT | EIF4A1 | GTPBP4 | BUB1B |
| COPS7B | ENG | H2AFZ | BUB3 |
| CPLX1 | ESAM | HSP90AA1 | BZW2 |
| CPNE7 | FAP | HSP90AB1 | C11orf73 |
| CPSF3 | FCER1G | HSPA8 | C12orf45 |
| CRABP2 | FCGR2A | HSPE1 | C16orf70 |
| CSE1L | FCGR3A | IARS | C17orf53 |
| CSNK1E | FILIP1L | IFITM1 | C19orf48 |
| CSNK2A2 | FKBP10 | IFITM2 | C1orf112 |
| CST1 | FN1 | IFITM3 | C1QBP |
| CTHRC1 | FNDC1 | IGFBP7 | C20orf196 |
| CTSK | FPR1 | IL23A | C20orf24 |
| CXCL10 | FSTL1 | ILF2 | C20orf27 |
| CXCL11 | FTL | IMPDH2 | C20orf96 |
| DAP3 | FXYD5 | ISY1 | C21orf59 |
| DARS | GBP1 | ITGAV | C2orf15 |
| DCLRE1B | GFPT2 | ITGB1BP1 | C5orf28 |
| DCTD | GNA12 | KIAA1143 | C5orf34 |
| DCUN1D5 | GNAI2 | KIAA1429 | C9orf114 |
| DDX10 | GNPDA1 | KPNA3 | C9orf116 |
| DDX21 | GPR137B | KPNB1 | CA9 |
| DDX31 | GPR176 | LDHB | CACYBP |
| DEGS1 | GPX1 | LSM1 | CAD |
| DFFA | GRAMD1A | LSM3 | CALR |
| DGAT2 | GRINA | LSM5 | CALU |
| DHX9 | GTF3A | LTA4H | CANX |
| DKC1 | HABP4 | LTV1 | CARM1 |
| DNAJC10 | HAPLN3 | LYAR | CASC5 |
| DNMT1 | HECW2 | MAGOH | CBX3 |
| DPP7 | HTRA1 | MATR3 | CCDC103 |
| DTD1 | ICAM1 | MBTPS2 | CCDC59 |
| DTX3L | IFITM1 | MCFD2 | CCDC74B |
| DUSP14 | IFITM2 | MCM3 | CCL26 |
| DUSP4 | IFITM3 | METTL5 | CCNA2 |
| E2F3 | IGFBP4 | MKRN2 | CCNB1 |
| E2F4 | IGFBP7 | MPV17 | CCND1 |
| ECT2 | IL23A | MRPL33 | CCNE1 |
| EDNRA | IL6 | MRPL36 | CCNF |
| EEF1E1 | ITGA5 | MRPL40 | CCT2 |
| EFTUD2 | KCNJ15 | MRPL45 | CCT3 |
| EIF2AK2 | KIRREL | MRPL51 | CCT4 |
| EIF4A1 | LAMC1 | MRPS18C | CCT6A |
| EIF4A3 | LEF1 | MTAP | CCT7 |
| ELK1 | LGALS1 | MTHFD1L | CCT8 |
| EMG1 | LILRB3 | MTR | CDC123 |
| ENC1 | LIMS1 | MYC | CDC20 |
| ENO1 | LMCD1 | MYL6B | CDC23 |
| EPHB4 | LOX | NAP1L1 | CDC25A |
| EPRS | LOXL1 | NKRF | CDC25B |
| ESM1 | LRRC15 | NOB1 | CDC25C |
| EXO1 | LRRC32 | NONO | CDC7 |
| EXOSC2 | LTBP2 | NSL1 | CDCA2 |
| EXTL2 | LUM | NUDCD2 | CDCA3 |
| FADS2 | M6PR | NUDT21 | CDCA5 |
| FAM60A | MFAP2 | NUDT5 | CDCA7 |
| FANCB | MMP1 | NUP133 | CDCA8 |
| FAP | MMP14 | NUP153 | CDH11 |
| FASTKD2 | MMP7 | NUP205 | CDH3 |
| FBL | MMP9 | PA2G4 | CDK2 |
| FEN1 | MNDA | PABPC1 | CDK4 |
| FNDC1 | MRC2 | PARK7 | CDK5 |
| FTSJ1 | MSC | PARP1 | CDK5RAP2 |
| FXN | MSTO1 | PATZ1 | CDKN3 |
| FXYD5 | MTHFD1L | PCID2 | CDR2L |
| GARS | MXRA5 | PCNA | CENPF |
| GART | NAP1L1 | PDCD2 | CENPH |
| GEMIN6 | NCF2 | PDCD5 | CENPI |
| GINS1 | NNMT | POLG2 | CENPN |
| GLO1 | NOTCH3 | POLR1A | CENPP |
| GMPS | NOX4 | POLR2D | CENPQ |
| GNB2L1 | NUAK1 | POLR2G | CEP55 |
| GNL3 | OLFML2B | PPA1 | CEP72 |
| GNPDA1 | OLR1 | PPT1 | CETN2 |
| GOLT1B | PAPSS1 | PRKRA | CFB |
| GPATCH4 | PCOLCE | PRPS1 | CGREF1 |
| GPR180 | PDGFRB | PSMA3 | CHAF1B |
| GRAMD1A | PDPN | PSMA5 | CHCHD6 |
| GRHL1 | PEA15 | PSMB1 | CHEK1 |
| GRIN2B | PLAU | PSMB4 | CHEK2 |
| GRIN2D | PLXND1 | PSMD10 | CHID1 |
| GRINA | PML | PSMF1 | CHPF |
| GRPEL2 | PPT1 | PTP4A3 | CIAPIN1 |
| GSTO1 | PRRX1 | PTPN12 | CKAP2 |
| GTF2H3 | PTAFR | PUM1 | CKAP5 |
| GTF3A | RAB31 | PWP1 | CKS1B |
| GTPBP4 | RAB34 | PYCR2 | CKS2 |
| HAPLN3 | RHOQ | RAI1 | CLDN1 |
| HEATR3 | RNF24 | RAN | CLDN2 |
| HM13 | RPL27 | RANBP1 | CLNS1A |
| HN1L | S100A8 | RBBP7 | CLPB |
| HOMER1 | S100A9 | RBX1 | CLPP |
| HPRT1 | SDCCAG3 | RFX5 | CLSPN |
| HSP90AA1 | SELE | RIPK2 | CMTM8 |
| HSP90AB1 | SERPINE1 | RMND1 | CNN2 |
| HSPA8 | SERPING1 | RNASEH2B | COL10A1 |
| HSPD1 | SFRP4 | RNF4 | COL12A1 |
| HSPE1 | SGIP1 | RP9 | COL1A2 |
| IARS | SH3PXD2B | RPA1 | COL5A1 |
| IFITM1 | SLC11A1 | RPL10A | COL8A1 |
| IFITM2 | SLC15A3 | RPL12 | COMMD2 |
| IFITM3 | SLC2A3 | RPL13 | COMT |
| IGFBP7 | SLC39A6 | RPL13A | COPS6 |
| IL23A | SLC43A3 | RPL14 | COPS7B |
| ILF2 | SLC7A5 | RPL15 | COPZ1 |
| IMMP2L | SNAI1 | RPL17 | CPLX1 |
| INTS7 | SNX10 | RPL19 | CPNE7 |
| IPO11 | SOD2 | RPL23 | CPSF3 |
| IRAK1 | SPARC | RPL23A | CPSF6 |
| ITGBL1 | SPOCK1 | RPL26 | CRLS1 |
| KARS | SPP1 | RPL27 | CSE1L |
| KDELC1 | SPRY1 | RPL30 | CSNK1E |
| KIAA1199 | SRM | RPL31 | CSNK1G2 |
| KIRREL | ST3GAL2 | RPL35A | CSNK2A2 |
| KLK6 | STAT1 | RPL37 | CST1 |
| KPNA3 | STC1 | RPL37A | CST2 |
| KRT80 | STX2 | RPL38 | CSTF1 |
| LARP1 | SULF1 | RPL39 | CSTF2 |
| LAS1L | SYDE1 | RPL7 | CTHRC1 |
| LGR6 | TGFB1 | RPL9 | CTNNBL1 |
| LPGAT1 | TGFB3 | RPS12 | CUEDC2 |
| LRP8 | THBS2 | RPS13 | CUL4A |
| LSM2 | THY1 | RPS14 | DAP3 |
| LSM5 | TIMP1 | RPS15A | DARS |
| LSM7 | TNC | RPS17 | DCLRE1A |
| LY6E | TNFAIP6 | RPS18 | DCTD |
| LYAR | TNFSF13B | RPS19 | DCUN1D5 |
| LZTS1 | TPST1 | RPS20 | DDX19A |
| MAPKAPK3 | TREM1 | RPS23 | DDX20 |
| MCFD2 | TRPV2 | RPS25 | DDX21 |
| MCM3 | UBE2L6 | RPS27A | DDX28 |
| MCM6 | VASH1 | RPS3 | DDX31 |
| MCM7 | VCAN | RPS5 | DDX49 |
| MID1IP1 | VEGFC | RPS6 | DDX56 |
| MIF | VKORC1 | RPS7 | DEGS1 |
| MMP1 | WARS | RPS8 | DEK |
| MMP11 | WISP1 | RSL1D1 | DEPDC1B |
| MMP7 | ZNF532 | SCRN1 | DFFA |
| MPV17 |  | SDAD1 | DGAT2 |
| MRPL17 |  | SEH1L | DHCR7 |
| MRPL36 |  | SET | DHODH |
| MRPL50 |  | SF3A3 | DHX30 |
| MRPL51 |  | SLC38A2 | DHX33 |
| MRPL9 |  | SLC39A6 | DHX9 |
| MRPS23 |  | SNRPB2 | DIAPH3 |
| MSTO1 |  | SNRPD2 | DKC1 |
| MSX1 |  | SNRPF | DLGAP4 |
| MTAP |  | SS18L2 | DNAJA1 |
| MTHFD1L |  | SSB | DNAJA3 |
| MTHFD2 |  | SSBP1 | DNAJC10 |
| MXRA5 |  | SSR1 | DNMT1 |
| MYC |  | SSRP1 | DNTTIP1 |
| MYL6B |  | ST13 | DNTTIP2 |
| NAP1L1 |  | STRAP | DOLK |
| NAT10 |  | STT3A | DPEP1 |
| NCL |  | SUMO2 | DPH2 |
| NDE1 |  | SYNCRIP | DPM1 |
| NFE2L3 |  | SYPL1 | DPM2 |
| NIP7 |  | TARDBP | DSN1 |
| NKD1 |  | TBCA | DTD1 |
| NKRF |  | TBCE | DTL |
| NME1 |  | TCEB1 | DTX3L |
| NMI |  | TFB2M | DTYMK |
| NOB1 |  | TGFBI | DUS1L |
| NOLC1 |  | TGIF2 | DUSP10 |
| NONO |  | THOC7 | DUSP14 |
| NOX4 |  | TIMM23 | E2F1 |
| NQO2 |  | TIMM9 | E2F3 |
| NUDCD1 |  | TMEM123 | E2F4 |
| NUDCD2 |  | TMEM14B | E2F5 |
| NUDT21 |  | TOMM20 | E2F7 |
| NUDT5 |  | TOMM7 | EBNA1BP2 |
| NUP133 |  | TOR1AIP1 | EBPL |
| NUP155 |  | TPT1 | ECT2 |
| NUP188 |  | TRIM32 | EDNRA |
| NUP205 |  | TTC27 | EEF1E1 |
| NUP37 |  | TTL | EFTUD2 |
| NUP62 |  | TUBE1 | EIF1AX |
| NUP85 |  | USPL1 | EIF2S2 |
| NXT1 |  | UTP18 | EIF4A1 |
| ODF2 |  | UTP6 | EIF4A3 |
| OGFOD1 |  | UXT | EIF5A |
| OLFML2B |  | VBP1 | EIF5A2 |
| PA2G4 |  | WDR36 | EIF5B |
| PAICS |  | XPO1 | ELK1 |
| PALB2 |  | XPO4 | EMD |
| PALM2 |  | YARS2 | ENC1 |
| PAPSS1 |  | YEATS2 | ENO1 |
| PCNA |  | YTHDF2 | EPB41L2 |
| PDCD2 |  | ZBTB2 | EPHB2 |
| PDCD2L |  | ZNF121 | EPHB3 |
| PDGFRB |  | ZNF2 | EPHB4 |
| PDPN |  | ZNF343 | EPRS |
| PEA15 |  | ZNF770 | ERAL1 |
| PFDN2 |  | ZZZ3 | EREG |
| PFDN4 |  |  | ESCO2 |
| PGM2 |  |  | ESM1 |
| PHLDA1 |  |  | ETAA1 |
| PLAU |  |  | ETV4 |
| PLS3 |  |  | ETV7 |
| PLXNA1 |  |  | EXO1 |
| PML |  |  | EXOSC2 |
| PODXL |  |  | EXOSC3 |
| POGK |  |  | EXOSC4 |
| POLD2 |  |  | EXOSC7 |
| POLR1A |  |  | EXOSC8 |
| POLR1C |  |  | EXTL2 |
| POLR1E |  |  | EZH2 |
| POLR2D |  |  | F2RL2 |
| POLR2G |  |  | FADS2 |
| PPA1 |  |  | FAIM |
| PPAT |  |  | FAM58A |
| PPIA |  |  | FAM60A |
| PPIH |  |  | FAM72A |
| PPIL1 |  |  | FAM83D |
| PPIL3 |  |  | FAM96B |
| PPP2R2A |  |  | FAM98A |
| PPRC1 |  |  | FANCA |
| PPT1 |  |  | FANCB |
| PRDX4 |  |  | FANCD2 |
| PRKDC |  |  | FANCG |
| PRMT1 |  |  | FAP |
| PRRX1 |  |  | FASTKD2 |
| PSAT1 |  |  | FBL |
| PSMA7 |  |  | FDPS |
| PSMB4 |  |  | FEN1 |
| PSMB9 |  |  | FIGNL1 |
| PSMC2 |  |  | FKBP7 |
| PSMD4 |  |  | FLAD1 |
| PSMF1 |  |  | FNDC1 |
| PTP4A3 |  |  | FOXM1 |
| PTPN11 |  |  | FOXQ1 |
| PUS7 |  |  | FTSJ1 |
| PWP1 |  |  | FTSJ2 |
| PYCR2 |  |  | FTSJ3 |
| QTRTD1 |  |  | FXN |
| R3HDM1 |  |  | FXYD5 |
| RAB36 |  |  | FZD3 |
| RAD23A |  |  | FZD6 |
| RAD51AP1 |  |  | G6PC3 |
| RAI1 |  |  | G6PD |
| RAN |  |  | GALNT6 |
| RANBP1 |  |  | GAPDH |
| RBM28 |  |  | GARS |
| RCC2 |  |  | GART |
| RCL1 |  |  | GDF11 |
| REXO2 |  |  | GEMIN5 |
| RGS16 |  |  | GEMIN6 |
| RHPN1 |  |  | GEMIN8 |
| RIOK1 |  |  | GINS1 |
| RIPK2 |  |  | GINS2 |
| RNASEH2A |  |  | GINS3 |
| RNASEH2B |  |  | GLA |
| RNF24 |  |  | GLO1 |
| RPA3 |  |  | GMPS |
| RPL12 |  |  | GNB2L1 |
| RPL23A |  |  | GNPDA1 |
| RPL27 |  |  | GOLT1A |
| RPL31 |  |  | GOLT1B |
| RPL35A |  |  | GORASP2 |
| RPL37A |  |  | GPATCH4 |
| RPL39 |  |  | GPI |
| RPS15A |  |  | GPR180 |
| RPS18 |  |  | GRAMD1A |
| RPS21 |  |  | GRHL1 |
| RQCD1 |  |  | GRIN2B |
| RSL1D1 |  |  | GRIN2D |
| RUNX1 |  |  | GRINA |
| RUVBL1 |  |  | GRPEL1 |
| S100A11 |  |  | GRPEL2 |
| S100A8 |  |  | GRWD1 |
| S100A9 |  |  | GSG2 |
| SCD |  |  | GSPT1 |
| SCLY |  |  | GSTO1 |
| SDAD1 |  |  | GSTP1 |
| SDCCAG3 |  |  | GTF2F2 |
| SELV |  |  | GTF2H3 |
| SEPHS1 |  |  | GTF2H5 |
| SET |  |  | GTF2IRD1 |
| SETD1A |  |  | GTF3A |
| SF3A3 |  |  | GTF3C4 |
| SF3B3 |  |  | GTPBP4 |
| SFXN3 |  |  | GYLTL1B |
| SH3PXD2B |  |  | H2AFY |
| SHMT2 |  |  | H2AFZ |
| SLC25A32 |  |  | HAT1 |
| SLC29A1 |  |  | HAX1 |
| SLC39A10 |  |  | HCFC1 |
| SLC39A6 |  |  | HDAC8 |
| SLC6A6 |  |  | HEATR3 |
| SLC7A5 |  |  | HELLS |
| SMARCC1 |  |  | HIST1H2AB |
| SMOX |  |  | HIST1H2AH |
| SNAI1 |  |  | HIST1H2BA |
| SND1 |  |  | HIST1H2BL |
| SNRPB2 |  |  | HIST1H2BO |
| SNRPD1 |  |  | HIST2H2AB |
| SNRPD2 |  |  | HIST3H2A |
| SNRPF |  |  | HM13 |
| SNRPG |  |  | HMGA1 |
| SNTB1 |  |  | HMMR |
| SORD |  |  | HN1L |
| SPARC |  |  | HOMER1 |
| SRM |  |  | HOXD9 |
| SRPRB |  |  | HPRT1 |
| SSB |  |  | HSD17B10 |
| SSBP1 |  |  | HSD17B12 |
| ST3GAL2 |  |  | HSP90AA1 |
| ST7 |  |  | HSP90AB1 |
| STAT1 |  |  | HSPA4 |
| STC2 |  |  | HSPA8 |
| STIP1 |  |  | HSPD1 |
| STRAP |  |  | HSPE1 |
| SULF1 |  |  | HSPH1 |
| SUV39H1 |  |  | HUNK |
| SYNCRIP |  |  | HYAL3 |
| TALDO1 |  |  | IARS |
| TAP1 |  |  | ICA1 |
| TCEB1 |  |  | ICT1 |
| TFDP1 |  |  | IFI6 |
| TGFBI |  |  | IFITM1 |
| TGIF2 |  |  | IFITM2 |
| THBS2 |  |  | IFITM3 |
| THY1 |  |  | IFRD2 |
| TIMM23 |  |  | IL15RA |
| TIMM9 |  |  | ILF2 |
| TIMP1 |  |  | IMP4 |
| TMEM109 |  |  | IMPDH2 |
| TMEM123 |  |  | INHBA |
| TMEM9 |  |  | INTS7 |
| TMPRSS3 |  |  | IPO11 |
| TMSB10 |  |  | IPO4 |
| TNFAIP6 |  |  | IPO7 |
| TOMM34 |  |  | IQGAP3 |
| TPD52L2 |  |  | IRAK1 |
| TRAP1 |  |  | ITGA2 |
| TRIB3 |  |  | ITGA6 |
| TRIM28 |  |  | ITGB3BP |
| TRIP13 |  |  | ITGBL1 |
| TRMT1 |  |  | ITPA |
| TSPAN5 |  |  | KARS |
| TTC27 |  |  | KBTBD6 |
| TTL |  |  | KDELC1 |
| TTYH3 |  |  | KDELR3 |
| TUBA1B |  |  | KIF11 |
| TUBA4A |  |  | KIF14 |
| TULP3 |  |  | KIF15 |
| TWISTNB |  |  | KIF18A |
| UBFD1 |  |  | KIF20A |
| UMPS |  |  | KIF23 |
| UTP14A |  |  | KIF2C |
| UTP18 |  |  | KIF4A |
| VASH2 |  |  | KIFC1 |
| VCAN |  |  | KIRREL |
| VSNL1 |  |  | KLF1 |
| WDR3 |  |  | KLHL18 |
| WDR36 |  |  | KLK6 |
| WDR4 |  |  | KPNA2 |
| WDR54 |  |  | KPNA3 |
| WDR66 |  |  | KRT80 |
| WDR75 |  |  | LAGE3 |
| WISP1 |  |  | LAPTM4B |
| XPO1 |  |  | LARP1 |
| XPO4 |  |  | LAS1L |
| XPO5 |  |  | LDHB |
| XPO6 |  |  | LGR5 |
| XRCC4 |  |  | LGR6 |
| YAP1 |  |  | LIN9 |
| YARS |  |  | LMNB2 |
| YARS2 |  |  | LPGAT1 |
| YEATS2 |  |  | LRP8 |
| YTHDF1 |  |  | LRRC8E |
| ZAK |  |  | LSM12 |
| ZBTB33 |  |  | LSM2 |
| ZNF121 |  |  | LSM5 |
| ZNF511 |  |  | LSM7 |
| ZYX |  |  | LY6E |
|  |  |  | LYAR |
|  |  |  | MAD2L1 |
|  |  |  | MAGED2 |
|  |  |  | MAGOH |
|  |  |  | MAP4 |
|  |  |  | MAPKAPK3 |
|  |  |  | MARS2 |
|  |  |  | MBTPS2 |
|  |  |  | MCFD2 |
|  |  |  | MCM10 |
|  |  |  | MCM2 |
|  |  |  | MCM3 |
|  |  |  | MCM6 |
|  |  |  | MCM7 |
|  |  |  | MCM8 |
|  |  |  | MELK |
|  |  |  | MET |
|  |  |  | METTL2B |
|  |  |  | METTL5 |
|  |  |  | MID1IP1 |
|  |  |  | MIF |
|  |  |  | MIPEP |
|  |  |  | MKI67 |
|  |  |  | MLF2 |
|  |  |  | MMP11 |
|  |  |  | MMP14 |
|  |  |  | MMP3 |
|  |  |  | MMP7 |
|  |  |  | MND1 |
|  |  |  | MORC4 |
|  |  |  | MPG |
|  |  |  | MPV17 |
|  |  |  | MPZL1 |
|  |  |  | MRPL15 |
|  |  |  | MRPL17 |
|  |  |  | MRPL18 |
|  |  |  | MRPL20 |
|  |  |  | MRPL22 |
|  |  |  | MRPL23 |
|  |  |  | MRPL3 |
|  |  |  | MRPL32 |
|  |  |  | MRPL36 |
|  |  |  | MRPL37 |
|  |  |  | MRPL4 |
|  |  |  | MRPL45 |
|  |  |  | MRPL47 |
|  |  |  | MRPL50 |
|  |  |  | MRPL51 |
|  |  |  | MRPL52 |
|  |  |  | MRPL55 |
|  |  |  | MRPL9 |
|  |  |  | MRPS12 |
|  |  |  | MRPS15 |
|  |  |  | MRPS2 |
|  |  |  | MRPS23 |
|  |  |  | MRPS24 |
|  |  |  | MRTO4 |
|  |  |  | MSTO1 |
|  |  |  | MTAP |
|  |  |  | MTBP |
|  |  |  | MTHFD1 |
|  |  |  | MTHFD1L |
|  |  |  | MTHFD2 |
|  |  |  | MYBBP1A |
|  |  |  | MYBL2 |
|  |  |  | MYC |
|  |  |  | MYCBP |
|  |  |  | MYEOV2 |
|  |  |  | MYL6B |
|  |  |  | MYT1 |
|  |  |  | N6AMT2 |
|  |  |  | NAT10 |
|  |  |  | NCAPD2 |
|  |  |  | NCL |
|  |  |  | NCLN |
|  |  |  | NDUFA4 |
|  |  |  | NDUFB11 |
|  |  |  | NEBL |
|  |  |  | NEK2 |
|  |  |  | NFATC3 |
|  |  |  | NFE2L3 |
|  |  |  | NIF3L1 |
|  |  |  | NIP7 |
|  |  |  | NKD1 |
|  |  |  | NKRF |
|  |  |  | NLE1 |
|  |  |  | NMB |
|  |  |  | NME1 |
|  |  |  | NME4 |
|  |  |  | NMI |
|  |  |  | NMU |
|  |  |  | NOB1 |
|  |  |  | NOLC1 |
|  |  |  | NOMO1 |
|  |  |  | NONO |
|  |  |  | NQO2 |
|  |  |  | NSDHL |
|  |  |  | NSUN2 |
|  |  |  | NT5DC2 |
|  |  |  | NT5DC3 |
|  |  |  | NTHL1 |
|  |  |  | NUDCD1 |
|  |  |  | NUDCD2 |
|  |  |  | NUDT1 |
|  |  |  | NUDT21 |
|  |  |  | NUDT5 |
|  |  |  | NUF2 |
|  |  |  | NUP133 |
|  |  |  | NUP155 |
|  |  |  | NUP188 |
|  |  |  | NUP205 |
|  |  |  | NUP37 |
|  |  |  | NUP43 |
|  |  |  | NUP85 |
|  |  |  | NUTF2 |
|  |  |  | NXT1 |
|  |  |  | ODF2 |
|  |  |  | OGFOD1 |
|  |  |  | OIP5 |
|  |  |  | OR2A12 |
|  |  |  | OTOF |
|  |  |  | OTUB2 |
|  |  |  | PA2G4 |
|  |  |  | PAFAH1B3 |
|  |  |  | PAICS |
|  |  |  | PAIP1 |
|  |  |  | PAK1IP1 |
|  |  |  | PALB2 |
|  |  |  | PALM2 |
|  |  |  | PAQR4 |
|  |  |  | PARK7 |
|  |  |  | PARP1 |
|  |  |  | PCBD1 |
|  |  |  | PCID2 |
|  |  |  | PCNA |
|  |  |  | PDCD2 |
|  |  |  | PDCD2L |
|  |  |  | PDCD5 |
|  |  |  | PDP2 |
|  |  |  | PEA15 |
|  |  |  | PES1 |
|  |  |  | PFDN2 |
|  |  |  | PFDN4 |
|  |  |  | PFDN6 |
|  |  |  | PGAM5 |
|  |  |  | PGK1 |
|  |  |  | PGM2 |
|  |  |  | PHF14 |
|  |  |  | PHF6 |
|  |  |  | PHLDA1 |
|  |  |  | PIGW |
|  |  |  | PIR |
|  |  |  | PITX1 |
|  |  |  | PKMYT1 |
|  |  |  | PLA2G2F |
|  |  |  | PLAU |
|  |  |  | PLK1 |
|  |  |  | PLOD3 |
|  |  |  | PLS3 |
|  |  |  | PLXNA1 |
|  |  |  | PMPCA |
|  |  |  | PNO1 |
|  |  |  | PODXL |
|  |  |  | POGK |
|  |  |  | POLD2 |
|  |  |  | POLE2 |
|  |  |  | POLR1A |
|  |  |  | POLR1C |
|  |  |  | POLR1E |
|  |  |  | POLR2C |
|  |  |  | POLR2D |
|  |  |  | POLR2E |
|  |  |  | POLR2I |
|  |  |  | POLR2K |
|  |  |  | POLR3F |
|  |  |  | POLR3G |
|  |  |  | POMP |
|  |  |  | POP1 |
|  |  |  | POP7 |
|  |  |  | PPA1 |
|  |  |  | PPAT |
|  |  |  | PPIA |
|  |  |  | PPIH |
|  |  |  | PPIL1 |
|  |  |  | PPME1 |
|  |  |  | PPP2R2A |
|  |  |  | PPRC1 |
|  |  |  | PQBP1 |
|  |  |  | PRDX1 |
|  |  |  | PRDX2 |
|  |  |  | PRDX4 |
|  |  |  | PREB |
|  |  |  | PRKAR1B |
|  |  |  | PRKDC |
|  |  |  | PRMT1 |
|  |  |  | PRMT5 |
|  |  |  | PROP1 |
|  |  |  | PRPS2 |
|  |  |  | PRR11 |
|  |  |  | PRSS22 |
|  |  |  | PSAT1 |
|  |  |  | PSMA2 |
|  |  |  | PSMA3 |
|  |  |  | PSMA5 |
|  |  |  | PSMA7 |
|  |  |  | PSMB1 |
|  |  |  | PSMB2 |
|  |  |  | PSMB3 |
|  |  |  | PSMB4 |
|  |  |  | PSMB5 |
|  |  |  | PSMB8 |
|  |  |  | PSMB9 |
|  |  |  | PSMC2 |
|  |  |  | PSMD13 |
|  |  |  | PSMD14 |
|  |  |  | PSMD2 |
|  |  |  | PSMD4 |
|  |  |  | PSMD7 |
|  |  |  | PSMD8 |
|  |  |  | PSMF1 |
|  |  |  | PTBP1 |
|  |  |  | PTGES3 |
|  |  |  | PTP4A3 |
|  |  |  | PTPN11 |
|  |  |  | PTRH1 |
|  |  |  | PTS |
|  |  |  | PTTG1 |
|  |  |  | PUS7 |
|  |  |  | PWP1 |
|  |  |  | PYCR1 |
|  |  |  | PYCR2 |
|  |  |  | QTRTD1 |
|  |  |  | RAB23 |
|  |  |  | RAB36 |
|  |  |  | RACGAP1 |
|  |  |  | RAD23A |
|  |  |  | RAD23B |
|  |  |  | RAD51 |
|  |  |  | RAD51AP1 |
|  |  |  | RAD51C |
|  |  |  | RAD54B |
|  |  |  | RAD54L |
|  |  |  | RAE1 |
|  |  |  | RAI1 |
|  |  |  | RAN |
|  |  |  | RANBP1 |
|  |  |  | RAP2A |
|  |  |  | RARG |
|  |  |  | RARS |
|  |  |  | RBBP9 |
|  |  |  | RBL1 |
|  |  |  | RBM28 |
|  |  |  | RCC2 |
|  |  |  | RECQL4 |
|  |  |  | REXO2 |
|  |  |  | RFC2 |
|  |  |  | RFC4 |
|  |  |  | RFC5 |
|  |  |  | RFWD3 |
|  |  |  | RGS12 |
|  |  |  | RGS16 |
|  |  |  | RHPN1 |
|  |  |  | RIOK1 |
|  |  |  | RIPK2 |
|  |  |  | RNASEH2A |
|  |  |  | RNASEH2B |
|  |  |  | RNF24 |
|  |  |  | RNF43 |
|  |  |  | RNPS1 |
|  |  |  | ROBO2 |
|  |  |  | RPA3 |
|  |  |  | RPE |
|  |  |  | RPL12 |
|  |  |  | RPL13 |
|  |  |  | RPL26L1 |
|  |  |  | RPL27 |
|  |  |  | RPL37A |
|  |  |  | RPL38 |
|  |  |  | RPL39 |
|  |  |  | RPL8 |
|  |  |  | RPLP0 |
|  |  |  | RPN2 |
|  |  |  | RPP40 |
|  |  |  | RPS15 |
|  |  |  | RPS15A |
|  |  |  | RPS16 |
|  |  |  | RPS18 |
|  |  |  | RPS19 |
|  |  |  | RPS21 |
|  |  |  | RPS7 |
|  |  |  | RQCD1 |
|  |  |  | RRM2 |
|  |  |  | RRP15 |
|  |  |  | RRP9 |
|  |  |  | RSL1D1 |
|  |  |  | RSRC1 |
|  |  |  | RTKN |
|  |  |  | RUVBL1 |
|  |  |  | RUVBL2 |
|  |  |  | S100A11 |
|  |  |  | S100A2 |
|  |  |  | SAE1 |
|  |  |  | SART3 |
|  |  |  | SCD |
|  |  |  | SCLY |
|  |  |  | SDAD1 |
|  |  |  | SDCCAG3 |
|  |  |  | SEC23B |
|  |  |  | SEC61B |
|  |  |  | SELV |
|  |  |  | SEPHS1 |
|  |  |  | SERBP1 |
|  |  |  | SERPINB5 |
|  |  |  | SERPINH1 |
|  |  |  | SET |
|  |  |  | SETD1A |
|  |  |  | SF3A3 |
|  |  |  | SF3B3 |
|  |  |  | SFXN3 |
|  |  |  | SGOL1 |
|  |  |  | SGOL2 |
|  |  |  | SH3BP4 |
|  |  |  | SH3TC2 |
|  |  |  | SHMT2 |
|  |  |  | SKP2 |
|  |  |  | SLBP |
|  |  |  | SLC12A2 |
|  |  |  | SLC12A8 |
|  |  |  | SLC19A1 |
|  |  |  | SLC1A4 |
|  |  |  | SLC22A12 |
|  |  |  | SLC25A15 |
|  |  |  | SLC25A32 |
|  |  |  | SLC29A1 |
|  |  |  | SLC35A2 |
|  |  |  | SLC35B4 |
|  |  |  | SLC39A10 |
|  |  |  | SLC39A6 |
|  |  |  | SLC4A11 |
|  |  |  | SLC5A6 |
|  |  |  | SLC6A6 |
|  |  |  | SLC7A1 |
|  |  |  | SLC7A5 |
|  |  |  | SLCO4A1 |
|  |  |  | SMAD5 |
|  |  |  | SMARCC1 |
|  |  |  | SMARCD1 |
|  |  |  | SMC2 |
|  |  |  | SMC4 |
|  |  |  | SMOX |
|  |  |  | SMYD5 |
|  |  |  | SNAI1 |
|  |  |  | SND1 |
|  |  |  | SNRPB |
|  |  |  | SNRPB2 |
|  |  |  | SNRPC |
|  |  |  | SNRPD1 |
|  |  |  | SNRPD2 |
|  |  |  | SNRPE |
|  |  |  | SNRPF |
|  |  |  | SNRPG |
|  |  |  | SNTB1 |
|  |  |  | SORD |
|  |  |  | SOX14 |
|  |  |  | SOX9 |
|  |  |  | SPAG5 |
|  |  |  | SPC25 |
|  |  |  | SQLE |
|  |  |  | SRM |
|  |  |  | SRP9 |
|  |  |  | SRPRB |
|  |  |  | SSB |
|  |  |  | SSBP1 |
|  |  |  | SSRP1 |
|  |  |  | SSSCA1 |
|  |  |  | SSX2IP |
|  |  |  | ST3GAL2 |
|  |  |  | ST7 |
|  |  |  | STC2 |
|  |  |  | STIP1 |
|  |  |  | STK3 |
|  |  |  | STMN1 |
|  |  |  | STOML2 |
|  |  |  | STRAP |
|  |  |  | STT3A |
|  |  |  | STXBP1 |
|  |  |  | SULF1 |
|  |  |  | SUMO1 |
|  |  |  | SUPT4H1 |
|  |  |  | SUV39H1 |
|  |  |  | SUV39H2 |
|  |  |  | SYNCRIP |
|  |  |  | SYNGR2 |
|  |  |  | SYPL1 |
|  |  |  | TAAR9 |
|  |  |  | TACC3 |
|  |  |  | TACSTD2 |
|  |  |  | TAF1A |
|  |  |  | TAGLN2 |
|  |  |  | TALDO1 |
|  |  |  | TARS2 |
|  |  |  | TBCA |
|  |  |  | TBL2 |
|  |  |  | TBRG4 |
|  |  |  | TCEB1 |
|  |  |  | TCF7 |
|  |  |  | TCOF1 |
|  |  |  | TDGF1 |
|  |  |  | TEAD1 |
|  |  |  | TEAD2 |
|  |  |  | TEAD4 |
|  |  |  | TESC |
|  |  |  | TFAM |
|  |  |  | TFB1M |
|  |  |  | TFB2M |
|  |  |  | TFDP1 |
|  |  |  | TG |
|  |  |  | TGFBI |
|  |  |  | THBS2 |
|  |  |  | THOC5 |
|  |  |  | THOC7 |
|  |  |  | THY1 |
|  |  |  | TIGD2 |
|  |  |  | TIMELESS |
|  |  |  | TIMM17A |
|  |  |  | TIMM23 |
|  |  |  | TIMM50 |
|  |  |  | TIMM9 |
|  |  |  | TIMP1 |
|  |  |  | TIPIN |
|  |  |  | TKT |
|  |  |  | TMEM109 |
|  |  |  | TMEM123 |
|  |  |  | TMEM147 |
|  |  |  | TMEM14A |
|  |  |  | TMEM41A |
|  |  |  | TMEM9 |
|  |  |  | TMEM97 |
|  |  |  | TMPO |
|  |  |  | TMPRSS13 |
|  |  |  | TMPRSS3 |
|  |  |  | TMSB10 |
|  |  |  | TNFSF15 |
|  |  |  | TNPO2 |
|  |  |  | TNS4 |
|  |  |  | TOMM20 |
|  |  |  | TOMM34 |
|  |  |  | TOMM40 |
|  |  |  | TOP2A |
|  |  |  | TPD52L1 |
|  |  |  | TPD52L2 |
|  |  |  | TPX2 |
|  |  |  | TRAIP |
|  |  |  | TRAP1 |
|  |  |  | TRIB3 |
|  |  |  | TRIM28 |
|  |  |  | TRIM29 |
|  |  |  | TRIM59 |
|  |  |  | TRIP13 |
|  |  |  | TRMT1 |
|  |  |  | TSN |
|  |  |  | TSTA3 |
|  |  |  | TTC27 |
|  |  |  | TTK |
|  |  |  | TTL |
|  |  |  | TTLL4 |
|  |  |  | TTYH3 |
|  |  |  | TUBA1A |
|  |  |  | TUBA1B |
|  |  |  | TUBA3C |
|  |  |  | TUBA4A |
|  |  |  | TUBB |
|  |  |  | TUBG1 |
|  |  |  | TULP3 |
|  |  |  | TWISTNB |
|  |  |  | U2AF1 |
|  |  |  | UBE2C |
|  |  |  | UBE2M |
|  |  |  | UBE2T |
|  |  |  | UBE2V1 |
|  |  |  | UBFD1 |
|  |  |  | UBIAD1 |
|  |  |  | UBQLN4 |
|  |  |  | UCHL3 |
|  |  |  | UCHL5 |
|  |  |  | UHRF1 |
|  |  |  | ULBP2 |
|  |  |  | UMPS |
|  |  |  | UNC5CL |
|  |  |  | URM1 |
|  |  |  | USP1 |
|  |  |  | USP14 |
|  |  |  | USP31 |
|  |  |  | USP5 |
|  |  |  | UTP11L |
|  |  |  | UTP14A |
|  |  |  | UTP15 |
|  |  |  | UTP18 |
|  |  |  | UTP20 |
|  |  |  | VARS |
|  |  |  | VBP1 |
|  |  |  | VCAN |
|  |  |  | VCP |
|  |  |  | VSNL1 |
|  |  |  | WDR12 |
|  |  |  | WDR18 |
|  |  |  | WDR3 |
|  |  |  | WDR36 |
|  |  |  | WDR4 |
|  |  |  | WDR5 |
|  |  |  | WDR62 |
|  |  |  | WDR66 |
|  |  |  | WDR77 |
|  |  |  | WDR90 |
|  |  |  | WISP1 |
|  |  |  | WNT3 |
|  |  |  | XPO1 |
|  |  |  | XPO4 |
|  |  |  | XPO5 |
|  |  |  | XPO6 |
|  |  |  | XRCC4 |
|  |  |  | XRN2 |
|  |  |  | YAP1 |
|  |  |  | YARS |
|  |  |  | YARS2 |
|  |  |  | YEATS2 |
|  |  |  | YTHDF1 |
|  |  |  | YWHAG |
|  |  |  | ZAK |
|  |  |  | ZBTB33 |
|  |  |  | ZDHHC9 |
|  |  |  | ZFHX2 |
|  |  |  | ZMYND19 |
|  |  |  | ZNF121 |
|  |  |  | ZNF239 |
|  |  |  | ZNF473 |
|  |  |  | ZNF511 |
|  |  |  | ZNF593 |
|  |  |  | ZNF598 |
|  |  |  | ZNF670 |
|  |  |  | ZNF687 |
|  |  |  | ZNRD1 |
|  |  |  | ZP3 |
|  |  |  | ZRANB3 |
|  |  |  | ZWILCH |
